# Feasibility and acceptability of hepatitis C virus self-testing models among high-risk groups in Nasarawa, Nigeria; Exploratory cross-sectional analysis of an implementation study

**DOI:** 10.1371/journal.pgph.0005567

**Published:** 2026-06-29

**Authors:** Victor Abiola Adepoju, Chidinma Umebido, Kristina Grabbe, Molly Strachan, Catharine Laube, Oluwafunke Odunlade, Jamiyu Ganiyu, Bashorun Adebobola, Ibrahim Adamu Alhassan, Adetiloye Oniyire, Cheryl Johnson, Karin Hatzold, Yasmin Dunkley

**Affiliations:** 1 Jhpiego Nigeria, Abuja, Nigeria; 2 Jhpiego, Baltimore, Maryland, United States of America; 3 World Health Organization, Abuja, Nigeria; 4 Federal Ministry of Health and Social Welfare, Abuja, Nigeria; 5 Nasarawa State Ministry of Health, Lafia, Nasarawa, Nigeria; 6 World Health Organization, Geneva, Switzerland; 7 Population Services International, Washington, District of Columbia, United States of America; 8 Department of Clinical Research, London School of Hygiene and Tropical Medicine, London, United Kingdom; PLOS: Public Library of Science, UNITED STATES OF AMERICA

## Abstract

Hepatitis C virus (HCV) infection remains a major public health challenge, with significant gaps in diagnosis and treatment in resource-limited settings. Hepatitis C self-testing (HCVST) offers a potential strategy to expand access, particularly in HIV clinical settings. We evaluated feasibility and acceptability of HCVST among high-risk populations in Nasarawa State, Nigeria when provided at antiretroviral (ART) clinics and one-stop shops (OSS) serving key populations (KP). 2,000 participants were enrolled between May 2023 and December 2023. Participants tested with either finger-prick blood-based or oral fluid antibody HCVST, and with or without health worker support. Follow-up documented results and linkage to qualitative RNA PCR confirmatory testing and treatment. Case-complete feasibility analyses were conducted across the HCV care cascade using chi-square tests (N = 1995). Acceptability was evaluated using a post-test survey score derived through case-complete factor analysis (N = 1868); associations between lowest decile acceptability score and participant characteristics were explored through regression analyses. Outcomes were compared by facility type (ART clinic vs. OSS). Free-text responses were thematically analyzed to contextualize findings. HCVST was feasible and acceptable. Of 226 reactive HCVST results (11.3%), 99.1% received RNA PCR testing. Among those with detectable RNA, 92% initiated treatment and 97% completed therapy. However, differences were observed by facility type. Participants in ART clinics were older, more likely to be female, and showed higher reactivity (15% vs. 8%) and treatment uptake (96% vs. 83%) than OSS clients. Acceptability was higher in ART clinics than OSS. HCVST was both feasible and acceptable in Nasarawa State, with some observed variations by facility type. These findings suggest that with differentiated service delivery models and adequate support for linkage, HCVST can increase HCV diagnosis, linkage to care, and treatment among high-risk groups in Nigeria, supporting integration of HCVST into national viral hepatitis elimination strategies.

## Introduction

Globally, an estimated 50 million people live with chronic hepatitis C virus (HCV) infection, and about 240,000 die each year from complications such as chronic liver disease, cirrhosis, and cancer [1]. While no vaccine exists, direct-acting antivirals (DAAs) now cure approximately 95% of cases [1–3], driving global momentum toward elimination. Yet major service gaps exist, particularly in low- and middle-income countries (LMIC). In 2022, only 36% of people with HCV globally knew their status, and just 20% of those diagnosed had initiated treatment [1,4].

HCV disproportionately affects vulnerable populations, especially people living with HIV (PLHIV) and key populations (KP), including people who inject drugs (PWID), men who have sex with men (MSM), and sex workers (SW) [5]. These groups face overlapping HCV and HIV risk factors such as needle sharing and condomless sex, compounded by stigma, discrimination, and legal barriers that limit care. WHO estimates 2.3 million or 5.9% of PLHIV have current or past HCV infection [6,7]. Sub-Saharan Africa bears the second-largest burden of HIV-HCV co-infection with around 430,000 cases [7]. Globally, more than half of co-infections occur among PLHIV who inject drugs [7]. PLHIV who are sex workers have 1.4-6.8 times higher odds of also living with HCV, while MSM and PWID face 4–13 times higher odds [7]. Co-infection accelerates liver disease progression, contributing significantly to morbidity and mortality [8].

Nigeria bears one of the highest HCV burdens, with approximately 2.4 million people chronically infected (1.1% prevalence) [9]. Prevalence peaks at ages 50–54 years (3.3%) and is lowest among 15–19-year-olds (0.4%) [9]. Among PLHIV, HCV prevalence is estimated at 1.1% [9], but some studies suggest rates up to 2.3% [10]. Data for key populations are limited but overlapping risks with PLHIV make them a priority for testing and treatment. The Nigerian epidemic is generalized with disproportionate burden among key populations. Awareness is critically low, over 80% of Nigerians with viral hepatitis do not know their status [11].

Nasarawa State carries a particularly high HCV burden, with an estimated 13.2% prevalence [9]—more than 250,000 people. In 2020 the state launched a five-year micro-elimination plan focusing on PLHIV and KP, but progress has been limited. By study initiation in 2023, only 85,000 people had been screened and 1,500 treated, far below the goal of testing 2.4 million and treating 124,000 by 2025 [12]. Expansion of HCV services has been constrained by high costs, stigma, and low public awareness. While HIV antiretroviral therapy (ART) clinics offer some capacity, community-based one-stop shops (OSS) for KP often lack HCV testing, limiting access for those most at risk.

HCV self-testing (HCVST) has emerged as a promising innovation to address these barriers. Endorsed by WHO in 2021, self-testing methods can expand access to HCV screening and enable higher rates of HCV treatment and cure among underserved populations [13]. Multi-country studies, including in Kenya and Egypt, found HCVST feasible and acceptable, with most users able to perform tests independently and willing to recommend them [14–19]. Benefits cited in those studies include privacy, convenience, and autonomy, while challenges included the need for confirmatory testing, linkage to care, and support to mitigate misuse or distress [20]. Finger-prick blood-based and oral fluid-based kits are available but not yet widely used in Nigeria, constrained by costs, regulatory barriers, and limited country-level evidence.

As Nigeria advances toward HCV elimination, especially in high-prevalence states like Nasarawa, context-specific evidence on HCVST is essential. Understanding feasibility and acceptability among PLHIV and KP can inform policy, drive down costs, and support scale-up. In particular, assessing HCVST in decentralized, non-clinical sites is critical, given gaps in current service delivery. This study examined the feasibility and acceptability of HCVST in ART clinics and OSS in Nasarawa State, Nigeria to guide future introduction and scale up.

## Methods

### Study design

This cross-sectional analysis of an implementation study explored feasibility and acceptability of HCVST among persons at high risk for acquiring HCV in Nasarawa State in Nigeria from 30/05/ 2023 to 30/04/2024 with recruitment conducted between 30/05/2023 to 04/12/2023. HCV care cascade outcomes were prospectively tracked throughout the implementation period. This manuscript follows the Strengthening the Reporting of Observational Studies in Epidemiology (STROBE) guidelines [21]. Additional exploratory qualitative analysis was conducted on free-text responses to enrich acceptability analyses.

### Setting

The study was conducted in Nasarawa State, selected due to the high prevalence of both HCV and HIV and the state’s commitment to addressing HCV as evidenced by efforts outlined in the HCV micro-elimination plan. Four sites were identified for participation in the study: two ART clinics serving PLHIV in public health facilities, and two community-based OSS serving KP. Sites were purposively selected based on populations served, level of care provided, and the presence of systems to provide follow-up services including linkage to confirmatory testing and HCV treatment.

### Study participants

The study enrolled 2,000 clients aged ≥18 years. Inclusion criteria were:

- Living with HIV and accessing care at participating ART clinics or,
- Seeking services at participating OSS, and
- with a negative or unknown HCV status at enrolment.

Clients were selected because their reported risk factors and service contexts place them at increased risk of HCV infection and co-infection and because they often face barriers to accessing conventional health services, including stigma, discrimination, and legal and social barriers. The sample size was determined by implementation research objectives and pragmatic recruitment considerations. A sample of 2000 participants permits estimation of an identified prevalence of 11.4% with a precision of approximately ±1.4 percentage points at the 95% confidence level.

### Implementation procedures

Clinicians in study sites were given a script to read to existing clients during routine clinic visits, health talks, or through routine community outreach. Clients interested in learning more about the study were introduced to a trained research assistant (RA) who described the study in detail. Once enrolled, study participants received health information on HCV transmission, prevention, and treatment availability, as well as instructions on how to conduct either finger-prick blood-based or oral fluid-based HCV antibody self-test, interpret the results and linkage procedures in the case of a reactive test. Professional HCV RDTs were available for all clients who were interested in HCV testing but did not consent to participate in the study although no enrolled participants opted for professional testing during the study period.

Participants could choose either a finger-prick blood-based lateral flow test (First Response HCV Card Test (Self-Test), Premier Medical Corps, Ltd., Gujarat, India) or an oral fluid-based rapid test (OraQuick HCV Self-Test, OraSure Technologies, Inc., Pennsylvania, USA). Once a test type was exhausted, participants could still enroll in the study but no longer had the option of choosing their test type. Because research-use supplies were finite, uptake differed by site and recruitment happened later in OSS sites than ART sites, finger-prick blood-based kits were depleted earlier through use in ART sites than oral-fluid kits. As a result, OSS participants enrolled after 13 November 2023 could only receive oral fluid–based HCVST.

Participants also had the option of conducting HCVST on-site in the presence of a health worker (observed), or on- or off-site without a health worker (unobserved). Those who chose unobserved HCVST could access on-demand assistance if they encountered difficulties, and were contacted by phone, text, WhatsApp, or home visit two days after receiving the HCVST to report their test results and support linkage to follow up services as necessary. A post-test survey to assess user experience was administered to all study participants.

The self-tests used in this study detect HCV antibodies (not viraemia) and therefore required confirmatory HCV RNA testing to diagnose current infection and determine treatment eligibility. Participants with a reactive HCVST were linked with a nearby health facility for an HCV RNA PCR test to confirm the presence of HCV and inform a treatment plan, as well as a liver function test, and clinical evaluation. Participants received up to five phone calls, messages, or home visits to support this linkage (days 3, 7, 14, 21, and 28). Those with detectable HCV RNA received treatment with DAAs (sofosbuvir/velpatasvir) per national guidelines, which was provided free of charge to all study participants. Drug abstention was not a prerequisite for confirmatory testing or treatment initiation, and treatment eligibility followed the national clinical pathway for HCV RNA-confirmed infection. Participants were followed up by phone throughout the course of treatment to confirm completion of DAAs and document cure as defined by the results of viral load testing done approximately 12 weeks post treatment. HCVST reactive study participants who also reported injecting drugs were offered the opportunity to engage their sexual or injecting drug use partners in the study—they were given a WhatsApp/SMS number to connect with study staff, who provided support and information about the study and HCV to the partners or referred them to a nearby HCV center for conventional testing and treatment if they preferred. Partners interested in participating in the study could pick up an HCVST from the study site, or directly from the initial study participant (secondary distribution).

### Exposure variables

Demographic, behavioral and clinical characteristics of study participants, as well as HCVST type used (finger-prick blood-based or oral fluid), approach (observed or unobserved), and facility type (ART clinic or OSS) were collected on an intake form administered upon study enrollment (**S1 Text**) and from an endline survey completed post-test (**S2 Text**).

Demographic variables collected were self-reported, grouped into: gender (male/female), age (18–24, 25–34, 35–44, 45–54 and 55+) education (no formal education, primary, high school, tertiary), occupational status (employed for wages, self-employed, out of work, student or “other”), marital status (single, never married, married/in domestic partnership, widowed, divorced/separated), referring to the highest level of education obtained to date. Data elements chosen aligned with national program data capture and retained sufficient statistical stability within groups.

Exposure variables hypothesized as associated with feasibility and acceptability were facility type (ART clinic or OSS), participant HIV status (positive, negative or unknown) and KP identity. KP identity was reporting any one of the following characteristics: persons who inject drugs (PWID—reported ever injected drugs for non-medical use), men having sex with men (MSM—reported male gender and same sex activity), sex worker (reported ever received goods or services for sex), and partners of sex workers (people who had ever paid for sex). Participants could report more than one key population identity. All variables were identified through self-report, with HIV status confirmed through clinical records where possible.

### Outcome variables

#### Feasibility.

Feasibility was explored through study uptake and care cascades; uptake of HCVST (study enrollment, use of HCVST), results (reactive, non-reactive), and—if reactive—access to follow up services (RNA PCR testing, treatment uptake, treatment completion and viral suppression) across all four sites. Case-complete feasibility analysis (N = 1995) was conducted. Only five participants were excluded from analysis as they were missing demographic and outcome data.

SVR12 outcomes were only available for participants who had reached 12 weeks post treatment by the end of data collection, and completed SVR12 testing, as follow up ended before all participants completed 12 weeks. The study did not systematically record the number or characteristics of clients who declined participation after receiving study information. As such, enrolment counts reflect uptake among eligible clients who consented to participate, rather than a population-level acceptance rate of HCV self-testing. A study logbook (**S3 Text**) tracked these follow-up service data as self-reported by participants and later verified with their HCV treatment center. Client data from the intake form was linked with that in the logbook using a unique identifier.

#### Acceptability.

Acceptability was explored through a composite client acceptability score constructed using exploratory and confirmatory factor analyses (EFA and CFA) derived from items from the endline satisfaction survey administered post-test which included structured Likert responses and free-text items. The survey was generated de novo for this study and had not been tested prior in this context. Data was entered in-person for those who completed the HCVST on-site, and by phone for those who used the test off-site.

Factor analysis was selected as the most appropriate method for the acceptability score given the latent, multidimensional nature of psychological acceptability constructs [22,23]. Seventeen indicators were identified as conceptually related to acceptability of HCV self-testing, drawing from four hypothesized domains: (i) ease of use, (ii) confidence and confidentiality, (iii) self-efficacy in managing care, and (iv) future use intentions. Items unrelated to the construct—such as HCV knowledge or willingness to pay—were excluded. All indicators used 5-point Likert response options, coded such that higher values reflected greater acceptability. Complete case analysis was used (N = 1868), given minimal item-level missingness.

Internal consistency for the full item pool was high (Cronbach’s α = 0.91), with sampling adequacy confirmed via the Kaiser-Meyer-Olkin test (KMO = 0.92) and Bartlett’s Test of Sphericity (p < 0.001), supporting factorability. EFA revealed a three-factor solution that aligned with initial conceptual domains (empowerment (5 items), ease of use (6 items), and future use intentions (3 items) except for the ‘confidence and confidentiality’ items, which showed low loadings and were excluded (3 items).

A 3-factor CFA was conducted on the remaining 14 indicators using the WLSMV estimator to account for ordinal-level Likert data. Model fit was good (CFI = 0.996; TLI = 0.995, RMSEA = 0.060; SRMR = 0.042). A second-order CFA specifying an overarching acceptability construct as a latent factor that explains shared variance across three first-order domains retained similarly strong fit indices (CFI = 0.996; TLI = 0.995; RMSEA = 0.060; SRMR = 0.042), supporting the validity of a single composite acceptability score. Participant-level factor scores were generated from the final model using regression (least squares). Given generally high rates of acceptability and left skew data distribution, and the need to characterise the minority of respondents with comparatively lower acceptability while retaining sufficient sample size for regression analyses, the continuous score was split into a binary outcome between the lowest decile acceptability score and the remaining acceptability scores for statistical analysis (**S4 Text**).

#### Qualitative exploration.

Qualitative thematic analysis of free-text responses to four open-ended questions in the endline survey (**S2 Text**) was conducted to provide additional insights and enrich quantitative cascade differences and acceptability. Free-text response questions elicited clients’ perspectives on preferences in terms of test-type, service location, and assistance modality as well as previous experiences with self-test technologies. A total of N = 1980 non-missing responses to at least one free-text item were recorded.

Responses were analysed using an exploratory, inductive thematic approach. Two members of the study team independently reviewed responses to develop an initial coding framework (MS and YD), which was iteratively refined through discussion. Coding focused on identifying recurring themes and contrasts by site type (ART clinic vs OSS) to contextualise observed differences in acceptability and cascade outcomes. Qualitative findings were used to enrich interpretation of quantitative results rather than to generate standalone explanatory theory.

### Statistical analysis

Data analysis was conducted in R studio and Excel for qualitative analysis.

Participant throughput was described; characteristics were described and compared by facility type using Pearson’s chi-square test with missingness described. Feasibility was explored through study enrollment, test results and linkage across the clinical care cascade, alongside differences by facility type (ART clinic vs. OSS), HIV status (people living with vs. without HIV) and KP identity (versus no reported KP identity). Due to very high collinearity between facility type, HIV status, and KP identity (almost all ART participants were PLHIV and non-KP; nearly all OSS participants were KP), facility type was retained as the sole exposure.

Acceptability was described as a continuous outcome and defined as a binary outcome (lowest decile acceptability score vs. acceptable). Continuous acceptability scores were explored visually using cumulative count plots and boxplots by age, gender, education status and facility type, with proportions in the lowest decile acceptability score identified. We modelled binary acceptability by facility type using logistic regression, adjusting for age, gender, and education status as a priori confounders. Qualitative themes were examined by site type (ART clinic vs. OSS) and integrated with quantitative findings to contextualise observed differences in cascade outcomes and acceptability.

### Ethics

Ethical approval for the study was obtained from the John Hopkins University Bloomberg School of Public Health Institutional Review Board (BPSH #20755), National Health Research and Ethics Committee in Nigeria (approval number NHREC/01/01/2007-17/07/2022), Nasarawa State Ministry of Health Ethics Review Committee (approval number NHREC 18/06/2017), and World Health Organization Ethics Review Committee (WHO ERC 3809). Study participants provided written informed consent.

## Results

### Participant throughput

**Fig 1:**
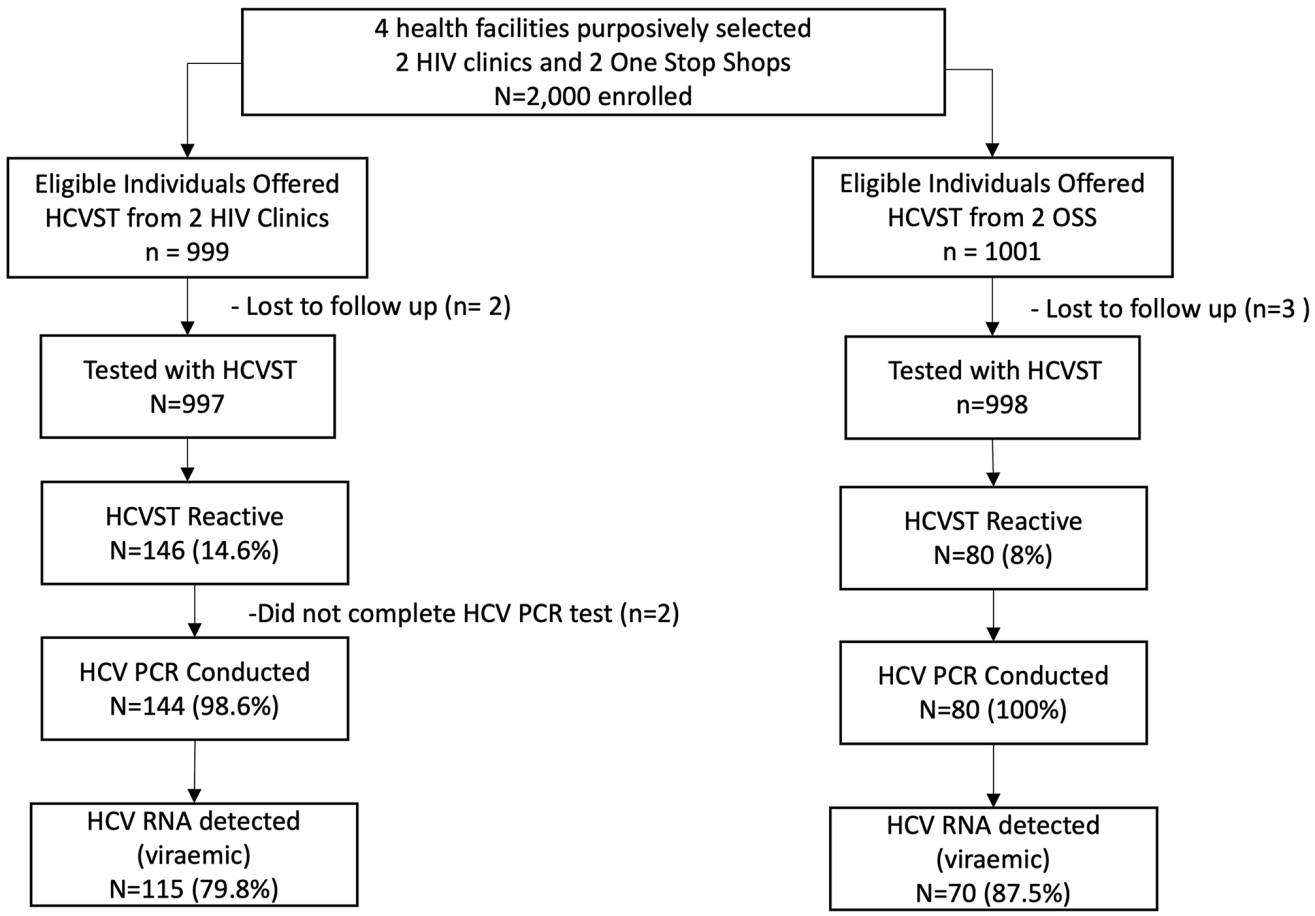
Study throughput.

A total of 2,000 people were enrolled and consented to participate in the study; five participants were missing demographic and outcome data and were excluded from feasibility analyses. The final analysis included data from 1,995 participants. 11 of these participants reported reactive HCVST results but did not complete the endline survey during the study period; their information is included and missingness described (**Fig 1**).

### Participant characteristics

#### Sociodemographic and behavioral factors.

Study participants were mostly female (60%), 25–44 years old (65%), and the majority had at least high school education (66%), were currently employed (66%), were not currently married or in a domestic partnership (53%), did not report KP identity characteristics (51%) and were living with HIV (65%). Of the 978 individuals reporting KP identity characteristics, only 16 (2%) were from an ART clinic, while 962 (98%) were from an OSS (Table 1). As a proportion of all OSS attendees (where participants could report multiple KP identities, 314 (32%) identified as MSM, 458 (46%) as sex workers, 279 (28%) as PWID, and 124 (12%) as partners of sex workers (**Table 1**).

**Table 1:** Participant characteristics.

Characteristics*	All sites N = 1,995 (%)	ART clinic N = 997 (50.0%)	OSS N = 998 (50.0%)	p-value^1^
**Gender identity**				
Male	799 (40.1)	240 (24.1)	559 (56.0)	<0.001
Female	1196 (59.9)	757 (75.9)	439 (44.0)	
**Age group**				
18-24	215 (10.8)	37 (3.7)	178 (18.0)	<0.001
25-34	675 (34.0)	191 (19.2)	484 (48.8)	
35-44	614 (30.9)	375 (37.8)	239 (24.1)	
45-54	322 (16.2)	253 (25.5)	69 (7.0)	
55+	158 (8.0)	137 (13.8)	21 (2.1)	
**Education status**				
No formal education	243 (12.2)	171 (17.2)	72 (7.3)	<0.001
Primary School	441 (22.2)	294 (29.6)	147 (14.8)	
High School	870 (43.9)	332 (33.4)	538 (54.3)	
Tertiary	430 (21.7)	196 (19.7)	234 (23.6)	
**Occupational status** ^2^				
Employed for wages	444 (22.4)	213 (21.5)	231 (23.3)	<0.001
Self-employed	862 (43.4)	324 (32.6)	538 (54.3)	
Out of work	265 (13.4)	126 (12.7)	139 (14.0)	
Student	75 (3.8)	23 (2.3)	52 (5.2)	
Retired/Other	328 (16.5)	305 (30.7)	23 (2.3)	
**Marital status**				
Single, never married	689 (34.7)	123 (12.4)	566 (57.1)	
Married/In domestic partnership	926 (46.7)	621 (62.5)	305 (30.8)	
Widowed	240 (12.1)	183 (18.4)	57 (5.8)	
Divorced/ Separated	129 (6.5)	66 (6.6)	63 (6.4)	
**Key Population identity characteristics**				
No reported identity characteristics	1006 (50.7)	977 (97.1)	29 (2.9)	<0.001
At least one key population identity characteristic reported (unique individuals)	978 (49.3)	16 (1.6)	962 (98.4)	
**- Of those reporting KP identity characteristics, breakdown by characteristic type** ^3^				
MSM	314	0 (0)	314 (31.5)	–
Sex Worker (SW)^4^	472	14 (1.4)	458 (45.9)	<0.001
PWID	279	0 (0)	279 (28.0)	–
Partners of SW	126	2 (0.2)	124 (12.4)	–
**HIV Status**				
Positive	1299 (65.1)	997 (100.0)	302 (30.3)	–
Negative	497 (24.9)	0 (0)	497 (49.8)	
Unknown	199 (10.0)	0 (0)	199 (19.9)	
**Test Modality**				
Observed	1135 (57.2)	574 (57.8)	561 (56.6)	0.622
Unobserved	849 (42.8)	419 (42.2)	430 (43.4)	
**Self-Test Type** ^**5**^				
Oral	965 (48.4)	377 (37.8)	588 (58.9)	<0.001
Blood	1030 (51.6)	620 (62.2)	410 (41.1)	

* Percentages are based on non-missing data; missing values: 11 participants did not complete Tool A, missing data therefore on age, education, occupational status, marital status, KP identity and breakdown of reported identity characteristics, and test modality. An additional 10 participants reported “not stated” for occupational status.

^1^Chi square test. If value is empty, insufficient parameter values for a statistically valid test.

^2^Other occupational status, n = 240 farmers.

^3^Reported identity characteristics; more than one identity characteristic possible per person, therefore column totals and percentages presented as a proportion of all attendees—however, combined identity characteristic totals will exceed 100%.

^4^Sex workers, of which female n = 418 (88.6%).

Most participants used the observed testing modality (57.2%), indicating they used the HCVST on-site with assistance from a health worker, whereas 42.8% used the test either on-site or off-site with no assistance from a health worker. This was the case in both the ART clinic and the OSS sites. A higher proportion of clients in OSS used oral-fluid HCVST (58.9%), compared to ART clinics where the majority of clients used blood-based HCVST (62.2%).

### Feasibility

**Fig 2:**
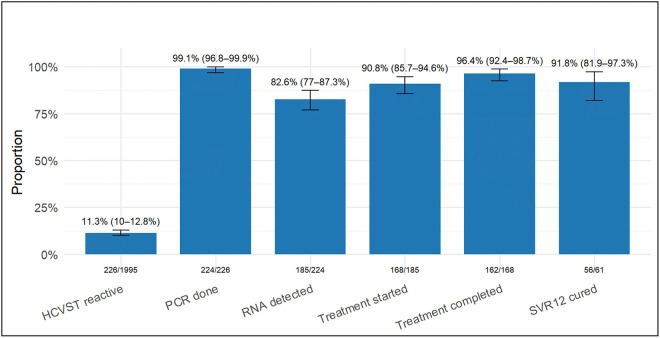
HCV clinical cascade outcomes; proportions (binomial exact confidence interval).

Feasibility was explored through study enrollment, test results and linkage across the clinical care cascade (**Fig 2**).

Among 1,995 participants who used the HCVST, 226 (11.3%) had reactive test results. Of those, 224 (99.1%) received an RNA PCR test, and HCV RNA was detected in 185 (82.6%). Among persons with RNA detected, 168 initiated treatment, corresponding to a treatment initiation rate of 90.8%. Treatment completion was documented in 163 participants (97.0% of those initiating therapy), and sustained virologic response at 12 weeks post-treatment (SVR12, indicative of cure) was achieved in 56 participants, representing 91.8% of those who underwent SVR assessment. Differences were assessed by facility type (ART clinic vs. OSS; **Table 2**).

**Table 2:** HCV clinical cascade by facility type.

Step	All Sites N (%)	ART Clinic N (%)	OSS N (%)	p-value*
Used HCVST	1995 (100%)	997 (100%)	998 (100%)	–
HCVST reactive	226 (11.3%)	146 (14.6%)	80 (8.0%)	<0.001
RNA PCR performed	224 (99.1%)	144 (98.6%)	80 (100%)	–
RNA detected	185 (82.6%)	115 (79.9%)	70 (87.5%)	0.207
Treatment started	168 (90.8%)	110 (95.7%)	58 (82.9%)	0.008
Treatment completed	163 (97.0%)	106 (96.4%)	56 (96.6%)	–
SVR12/ Cured^1^	56/61 (91.8%)	36/36 (100%)	20/25 (80.0%)	–

*Chi square test, values missing if any cell in the 2x2 table <5.

^1^Denominator for those achieving SVR12 includes only those participants who returned for viral load test and had available data at the end of the data collection period

Among individuals who used HCVST, the proportion with reactive test results was significantly higher at ART clinics compared to OSS (14.6% vs. 8.0%, *p* < 0.001). Among those with HCV RNA detected, treatment initiation was also higher at ART facilities, with 95.7% of RNA-positive individuals starting therapy compared to 82.9% at OSS facilities (*p* = 0.008).

### Acceptability

**Fig 3:**
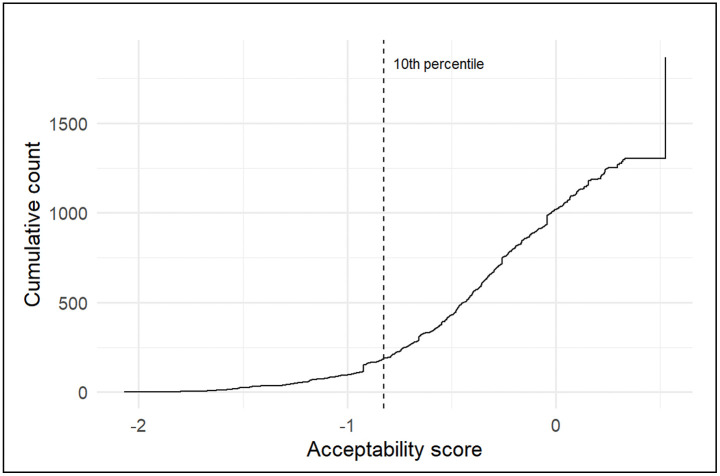
Cumulative count plot of acceptability score; values to the left of the dotted line indicate responses in the lowest decile acceptability score.

Continuous acceptability scores were explored visually using cumulative count plots and boxplots by age, gender, education status and facility type, with proportions in the lowest decile acceptability score identified. HCVST was highly acceptable in this sample, with only a small minority indicating lower acceptability across empowerment, ease of use, and future use intentions (**Fig 3**).

**Fig 4:**
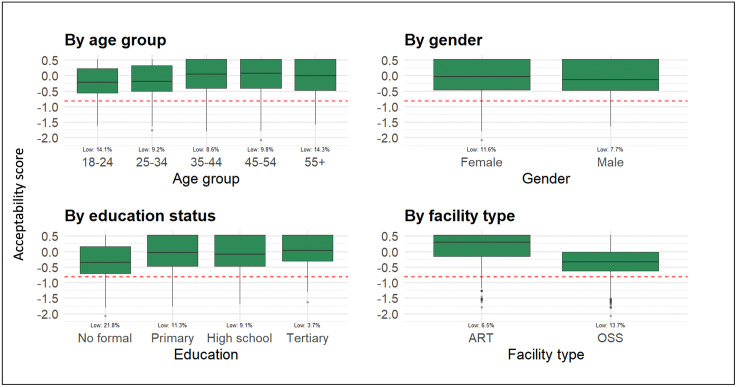
Box-and-Whisker plots of acceptability distribution by key covariates (age, gender, education, and facility type); Values below the dotted red line indicate responses in the lowest decile acceptability score. Proportions under this threshold are reported with corresponding percentages.

Visible differences in distributions were observed by facility type, educational status and age, while differences by gender were minimal. OSS site attendance had lower acceptability scores, as did lowest educational status (no formal education) and younger age groups (18–24, and 25–34) had lower acceptability scores. The red cutoff line highlighted the proportion of participants falling within the lowest decile acceptability score in each group (**Fig 4**).

We modelled binary acceptability by facility type using logistic regression, adjusting for age, gender, and education status as priori confounders. Modelling facility type as the primary exposure in unadjusted analysis, participants at OSS sites had 2.3 times the odds of being in the lowest decile of acceptability compared to those at ART clinics (OR = 2.3; 95% CI: 1.7–3.2; *p* < 0.001). After adjusting for age, sex, and education level, the association strengthened (aOR = 4.2; 95% CI: 2.8–6.2; *p* < 0.001) (**Table 3**).

**Table 3:** Odds of being in the lowest decile of acceptability by facility type (N = 1,868).

Facility Type	Unadjusted OR (95% CI)	Adjusted OR (95% CI)	p-value*
OSS	2.3 (1.7 to 3.2)	4.2 (2.8 to 6.2)	<0.001
ART Clinic	–	–	

*Wald test.

Qualitative analysis provided some potential explanatory detail both on the lower initiations of treatment at OSS and potentially lower acceptability. ART clients expressed a desire for more support when trying the self-tests but once trained, expressed very high confidence to use HCVST alone. They expressed a desire to “compare,” “be knowledgeable,” and “avoid mistakes,” suggesting a clear pathway from their initial need for assistance to independence. In contrast, participants in OSS were more likely to express a desire to use the HCVST alone, with some recognition that provider assistance was preferential, but only after attempting the test themselves. Attendees wanted to “just try something different”. As relates to feasibility, therefore, ART attendees appeared more likely to take the support offered in the clinic, whereas OSS attendees wanted to go it alone. This may reflect differences in clinic experience and engagement with formal health services on behalf of ART attendees that could be reflected in higher initiations on treatment once diagnosed.

However, acceptability appeared to be more associated with client characteristics than facility type: younger females (18–24 years) in the OSS expressed more concerns about needles and pain, as well as the highest initial need for guidance, and were less confident than their male counterparts. For example, “I will like to do it, in case I make a mistake, in the facility someone can guide me” (F, 18–24).

## Discussion

This study provides important insights into the feasibility and acceptability of HCVST among PLHIV and KP in Nasarawa State, Nigeria. HCVST emerged as feasible and acceptable in these study populations. Feasibility was reflected by high uptake of services across the clinical cascade among study participants. Nearly all participants with reactive HCVST results accessed confirmatory RNA testing (99%), and of those with detected RNA, 90.8% started treatment. Treatment completion rates were also high (97%), and for those who completed therapy and had a viral load test by the time the study ended, 92% achieved sustained virologic response (cure). These outcomes indicate that HCVST is consistent with progression through the clinical cascade when accompanied by supportive linkage systems. HCVST acceptability scores were also high among study participants.

Nevertheless, a critical observation in this study was the marked difference in treatment initiation and acceptability of the HCVST intervention based on facility type. Treatment initiation rates were significantly higher in ART clinics compared to OSS (95.7% vs. 82.9%) and acceptability scores were also higher. This may suggest that established ART clinics that are already embedded in formal health systems may offer more effective patient navigation and support for linkage to care than OSS, or they may reflect unmeasured factors, such as staffing levels, on-site pharmacy support, or same-day drug availability. This is conceptually plausible: patients in ART clinics are likely more clinically experienced than attendees of OSS, more used to formal health services, and more likely to accept clinician support to link into treatment. This is underlined by the findings of Sekhon et al., who posited that perceptions of acceptability of a healthcare intervention are influenced by participants’ and providers’ understanding of that intervention and how it works in relation to the problem it targets (“coherence”) [24]. Additional weight is given to this argument by the clear link from supportive testing intervention to independence amongst ART attendees in the qualitative results in this study. Although critical entry points for key populations, OSS may require strengthened referral and navigation systems to sustain downstream care, a finding with practical implications for program design and service integration.

This aligns with evidence from other LMIC contexts showing that decentralized or community-based facilities can successfully distribute self-tests but require additional attention for linkage to treatment [25,26]. Our findings are consistent with early studies on the introduction of HIV self-testing, which also showed generally positive findings and the need to tailor specific aspects—like linkage to treatment—for key populations in particular [27], and with a recent survey of Nigerians 18 years and above that showed generally strong support for HCVST among health workers and non-healthcare workers in Nigeria [28].

However, it is also important not to overstate these differences: lower acceptability and treatment may also reflect the structural difference in populations by site; OSS clients were generally younger and male compared to the ART sites; these populations are less likely to engage and be retained in care [29]. Differences in acceptability may also reflect both true differences in perceptions or potential differences in how different participants across settings interpreted or responded to scale items.

Finally, the proportion of participants identifying as PWID within OSS attendees (28%) was lower than anticipated given their broader HCV burden and the amount of harm reduction programming offered through the OSS. Nasarawa State has high HCV prevalence and strong outreach with waitlists for treatment, so it is possible that many PWID with known HCV status had already been reached.

### Strengths and limitations

Strengths of this study include the large sample size, a diverse mix of high-risk populations from ART clinics and OSS, smooth integration of HCVST into routine health services in the study sites, robust support for linkage to near point-of-care RNA PCR testing and free treatment, and the use of both quantitative and qualitative measures to assess feasibility and acceptability of HCVST.

The variability in sociodemographic characteristics and HCV reactivity across settings might reflect a selection bias in the study population, driven by the demographics of attendees at ART clinics and OSS. Moreover, acceptability findings in this study reflect perceptions and experiences among participants who consented to HCVST and completed post-test assessments. Because refusal rates were not systematically captured, we cannot estimate the proportion of all eligible clients who declined participation. Therefore, high enrolment and acceptability scores should not be interpreted as indicating universal acceptability of HCVST among all clinic or OSS attendees, but rather as evidence that HCVST is highly acceptable among those willing to engage with self-testing when offered.

Importantly, the acceptability measure used in this study was generated de novo and had not been tested prior in this context. Although there was high internal consistency and the tool demonstrated a coherent factor structure with good model fit under exploratory and confirmatory factor analyses, this approach was intended to identify latent structure within acceptability-related survey items rather than to develop or validate a formal psychometric instrument. It therefore remains plausible that, without prior validation, the constructs identified may not align fully with established theoretical frameworks of acceptability and instead reflect empirical response domains within this implementation context.

Additionally, we did not assess whether the acceptability scale functioned equivalently across ART and OSS settings, so observed differences should be interpreted with that caveat; no assurances can be made that this scale will function equivalently in different contexts.

Given the high HCV prevalence in Nasarawa we expected to see more PWID among the KP at OSS in particular, and the low number of PWID may reflect a discomfort in revealing PWID identity even in KP-friendly spaces. Furthermore, although the same set of questions was used across all sites to identify KP identity characteristics, very few ART clinic participants reported KP identity characteristics. This is not unexpected, as health facilities generally have low rates of self-reported KP identity due to stigma and discrimination [30]. Test type preference differed by service context, with OSS participants more frequently selecting oral-fluid tests. Given only oral-fluid tests were available after 13 November 2023, when ART recruitment had concluded and OSS recruitment was ongoing, this supply-related constraint likely contributes to the higher proportion of OSS participants using oral-fluid HCVST compared with ART clinics. Lastly, our study was not designed to capture SVR12 outcomes for all participants—the SVR12 data reported in our care cascade are incomplete because follow up ended before the 12-week time frame passed for all participants.

## Conclusion

Overall, the findings from this evaluation indicate high feasibility and broadly positive acceptability among study participants, particularly in ART clinics, and suggest that the broader introduction and scale up of HCVST in Nigeria could expand HCV diagnosis and treatment. HCVST integration in ART clinics may be particularly important for case finding and due to their successful linkage to treatment. However, targeted efforts are needed for OSS serving key populations and facilities with limited health system integration, including patient navigation, peer outreach, and stigma reduction.

Future research should aim to dissect the interrelated influences of key population identity, HIV status, and facility type, and further explore tailored interventions that address the unique needs and barriers faced by OSS users. Attention to qualitative insights will be crucial to inform strategies that enhance both feasibility and acceptability across diverse settings.

## Supporting information

S1 Text HCV study screening intake form. (PDF)

S2 Text Endline survey completed post-test. (PDF)

S3 Text Study logbook tracking HCV clinical outcomes. (PDF)

S4 Text Factor analysis for the acceptability score. (DOCX)

S1 Checklist Inclusivity in global research questionnaire. (DOCX)

S1 Data Supporting data. (XLSX)
