# Supplementary material for: Feasibility and acceptability of hepatitis C virus self-testing models among high-risk groups in Nasarawa, Nigeria; Exploratory cross-sectional analysis of an implementation study
Source: PLOS Glob Public Health. 2026 Jun 29;6(6):e0005567. doi: 10.1371/journal.pgph.0005567 (PMC13313356; doi:10.1371/journal.pgph.0005567)
Supplement: S1 Text — (PDF) [file pgph.0005567.s001.pdf]

HCV Study Screening Intake Form

The following will be asked by a research assistant prior to the use of the HCV Self Test:

- Name
- Address
- Mobile #1
- Mobile #2

Eligible

Consent

Intake information Prior to HVC Self-Test

Population Group:

Sex Worker:

Do you ever receive money or goods in exchange for sex?
Did you receive drugs in exchange for sex in the last one month?
Thinking of all the money you made last month, what percent would you estimate did you receive in exchange for sex?

Partner of a Sex Worker

Did you pay for sex in the last 12 months?

Men who have sex with Men

Have you ever had a sexual relationship with someone of same sex with you?

Transgender

Do you identify with a gender that does not conform with the sex you were assigned at birth?

Person Who Injects Drugs

Do you ever use drugs by injecting (using needles) for non-medical use?

Definitions:
FSW: Female receive money, goods or favors in exchange for sexual services, as primary source of income
MSM Sex Worker: MSM receive money, goods or favors in exchange for sexual services, as primary source of income
MSM: Men who engage in sexual relations with other men regardless of the motivation
PSW: Male/Female adults, who paid for sex in the last 12 months
Transgender: Male or female whose gender identity/expression does not conform with the sex assigned at birth
PWID: Female or male adult and young person who has injected at any time within the past 12 months any type of drug for non-medical use

HIV History:

Ever tested for HIV? Yes/No
Latest test date __/__/____
Result of HIV test done in the last 6 months,
HIV Status Positive Negative Unknown
Have you ever used an HIV self test?

ART clinic: date of ART initiation

Hepatitis C Virus History

Have you ever been diagnosed with hepatitis C virus?
If yes, what month and year were you diagnosed
What month did you start treatment?
Did you complete the 3 month course of treatment?
What month did you complete treatment?

Facility Type:

- HIV Care & treatment
- One Stop Shop
- Secondary Distribution

Test Type Selected

- Oral
- Blood based

Option selection:

- Assisted
- Unassisted
