# Supplementary material for: Feasibility and acceptability of hepatitis C virus self-testing models among high-risk groups in Nasarawa, Nigeria; Exploratory cross-sectional analysis of an implementation study
Source: PLOS Glob Public Health. 2026 Jun 29;6(6):e0005567. doi: 10.1371/journal.pgph.0005567 (PMC13313356; doi:10.1371/journal.pgph.0005567)
Supplement: S2 Text — (PDF) [file pgph.0005567.s002.pdf]

Tool A: Study Participant (Client) Questionnaire for Clients who used the HCV Self Test

Study ID_______

Demographic Information

1. Sex at birth: Male ☐ Female ☐
2. How old are you: _______
3. State of residence:
4. LGA of residence:

5. What is the highest degree or level of school you have completed? *If currently enrolled, highest degree received.*
   a) No schooling ☐
   b) Primary School ☐
   c) Secondary School ☐
   d) High school ☐
   e) Tertiary ☐

6. Marital Status: What is your marital status?
   a) Single, never married ☐
   b) Married or domestic partnership ☐
   c) Widowed ☐
   d) Divorced ☐
   e) Separated ☐

7. Employment Status: Are you currently…?
   a) Employed for wages ☐
   b) Self-employed ☐
   c) Out of work and looking for work ☐
   d) Out of work but not currently looking for work ☐
   e) A student ☐
   f) Retired
   g) Other (Please specify) ______________________

8. Have you ever used a self test kit of any kind? Yes/No
   If yes, type: pregnancy, COVID, HIV, HCV, other?

9. Do you have any thoughts you would like to share about the self tests you've taken in the past? (please describe likes and dislikes)?

Experiences Using Self Test Kit

		Strongly Agree	Agree	Neutral	Disagree	Strongly Disagree
**HCV attitudes**						
10.	I knew about HCV before I learned about this study.					
11.	HCV is a health concern that people in my community worry about.					
12.	I worry about getting HCV.					
13.	I had conversations about HCV with other people before I learned about this study.					
14.	It is comfortable to talk about HCV in my community.					
**Ease of use of HCVST**						
		Very Easy	Easy	Neither easy nor difficult	Difficult	Very Difficult
15.	HCV self-testing is_________					
16.	HCVST sample is _____to collect					
17.	If you used a blood-based test:<br>It was ____ to prick my finger (If you did not use a blood based test, skip to next question.)					
18.	It is ______ to use the HCVST self-test					
19.	The Instructions were _____to follow					
20.	HCVST result is ____ to interpret					
21.	It was _____ to report my self test results to the provider					
		Strongly Agree	Agree	Neutral	Disagree	Strongly Disagree
22.	HCV self-testing is private and confidential					
~~23.~~	~~I was able to get the privacy I needed to take the test~~					

24.	I am confident the HCVST results are accurate					
~~25.~~	~~I was able to use the test with no pain or discomfort~~					
26.	I prefer to self test than have a provider administer a test					
**Self Care:** Self-tests are a way of promoting self care which is defined as a "conscious act a person takes in order to promote their own physical, mental, and emotional health."						
27.	I feel that I can self test according to the instructions					
28.	I feel that I can understand safe use and can comply, and knows when to seek assistance					
29.	I feel that I can access information about self testing and express needs					
30.	I feel that I can exercise choice over whether or not to choose a self test					
31.	I feel that I can accurately determine when additional care is needed, know who and how to access support, and have ability to access that follow-up when needed					
**Linkage to confirmatory testing and treatment** *(for participants with reactive tests only)*						
32.	It was convenient to visit the provider for additional tests					
33.	The provider explained my test results clearly					
34.	*(for participants confirmed HCV-positive only)* It was convenient to access treatment.					
**Future use of HCVST**						
35.	I will recommend HCV self-test to my family and friends					
36.	I will take the HCVST kits home for my family and friends					
37.	I would use the HCVST again (if needed)					

Willingness to pay for HCVST						
38.	HCVST kits should be made available for purchase in public retail outlets					
39.	I would use HCVST if I had to pay for it.<br><br>I would be will to pay ___(read responses below) for an HCVST.<br><br>Free<br>Naira 500 – 1000<br>Naira 1000-1500<br>Naira 1500+					

40. If you were to use an HCV self test again would you prefer a blood or oral fluid based test?
    Blood __
    Oral fluid __
    No preference ___
    Unsure___

    Please explain why.

41. If you were to use a self test again would you prefer to use the test at home or at a health facility?
    Home __
    Health facility ___
    No preference ___
    Other preferred location__________

    Please explain why.

42. If you were to use a self test again would you prefer to do it yourself (unassisted) or to have a provider assist you in performing the test?
    Unassisted ___
    Assisted ___
    No preference ___
    Unsure____

    Please explain why.

43. What made you interested in taking an HCV self test? (check all that apply, rank 1-7 1= main reason; 7 = lowest reason)

    Desire to know HCV status
    Test was free
    Short Turn around Time

It is empowering to be able to test myself
I prefer privacy of testing on my own
I don't trust providers
Providers judge me/I feel stigmatized
Other feelings or reasons for using the self test: ______________

44. Did you prefer to keep your HCV self test experience private or did you tell a partner/family member/friend that you were taking a test?

    Prefer to keep private
    Tell partner/family/friend

Questions for participants with reactive tests only

45. Based on the verbal and written instructions you received, did you understand clearly what to do after you had a reactive (positive) self test? If not, please explain

    Refers to questions 53-55: After having a reactive test, you visited a health facility for additional tests by healthcare providers, and then you received your results on a different day. Please answer the following questions about your experience.

46. What did you like about this experience?

47. What did you not like about this experience?

48. What would you change about this experience if you could?
