# Supplementary material for: Feasibility and acceptability of hepatitis C virus self-testing models among high-risk groups in Nasarawa, Nigeria; Exploratory cross-sectional analysis of an implementation study
Source: PLOS Glob Public Health. 2026 Jun 29;6(6):e0005567. doi: 10.1371/journal.pgph.0005567 (PMC13313356; doi:10.1371/journal.pgph.0005567)
Supplement: S3 Text — (PDF) [file pgph.0005567.s003.pdf]

## Study Log book

| Date | Study Participant ID | Referral code (if provided) | Gender M/F/TG | Consent Y/N | Population Group FSW, MSM, PSW, TG, PWID, GenPop | HIV history Pos/Neg/ Unknown | If HIV+, ART Start Date | HCV history Pos/Neg/ Unknown | Type of Test Oral/Blood | Model Assisted/ Unassisted | Reactive Y/N | If yes, link to lab to treatment Date | what about partners? Secondary distribution |
|---|---|---|---|---|---|---|---|---|---|---|---|---|---|
| | | | | | | | | | | | | | |
| | | | | | | | | | | | | | |
| | | | | | | | | | | | | | |
| | | | | | | | | | | | | | |
| | | | | | | | | | | | | | |
| | | | | | | | | | | | | | |
| | | | | | | | | | | | | | |

## HCV Reactive Logbook

| Date | Study Participant ID | Date tested reactive | Date linked to lab/Facility | Confirmatory test date | Negative/Positive | If positive, LFT | LFT Result | Initated HCV treatment | F/U Call 1 | F/U Call 2 | F/U Call 3 | completed HCV treatment | sustained virologic response |
|---|---|---|---|---|---|---|---|---|---|---|---|---|---|
| | | | | | | | | | | | | | |
| | | | | | | | | | | | | | |
| | | | | | | | | | | | | | |
| | | | | | | | | | | | | | |
| | | | | | | | | | | | | | |

## Follow-up Tracking Unassisted Logbook

| Study Participant ID | Follow up 1 date | Outcome 1 | Follow up 2 date | Outcome 2 | Follow up 3 date | Outcome 3 | Completed questionnaire date | Not found |
|---|---|---|---|---|---|---|---|---|
| | | | | | | | | |
| | | | | | | | | |
| | | | | | | | | |
| | | | | | | | | |
| | | | | | | | | |
| | | | | | | | | |
| | | | | | | | | |
