# Supplementary material for: Feasibility and acceptability of hepatitis C virus self-testing models among high-risk groups in Nasarawa, Nigeria; Exploratory cross-sectional analysis of an implementation study
Source: PLOS Glob Public Health. 2026 Jun 29;6(6):e0005567. doi: 10.1371/journal.pgph.0005567 (PMC13313356; doi:10.1371/journal.pgph.0005567)
Supplement: S4 Text — (DOCX) [file pgph.0005567.s004.docx]

**Supplement 4. Factor analysis for the acceptability score.**

1. **Indicator List and Response Coding**

Table S4.1 presents the 17 Likert-scale items that were considered for inclusion in the acceptability construct, grouped by four conceptual domains identified a priori from the literature and formative work.

The four hypothesised domains reflected conceptually distinct but related dimensions of acceptability, derived from existing literature on self-testing acceptability frameworks. These were:

1. Ease of use (6 items; ac1–ac7), capturing perceptions of technical simplicity and clarity of instructions – Nb. ac3 was not included and is not presented here.
2. Confidence and confidentiality (3 items; ac8–ac10), assessing privacy, trust in results, and preference for self-testing over provider-administered testing.
3. Self-empowerment (5 items; ac11–ac15), representing self-efficacy, autonomy, and perceived control in testing and linkage; and,
4. Future use intentions (3 items; ac16–ac18), reflecting willingness to recommend, reuse, or distribute tests to others.

Each item used a 5-point response scale, coded such that higher scores reflect greater perceived acceptability. These indicators formed the initial item pool for psychometric testing.

**Table S4.1. 17 Likert-scale items considered for inclusion in the acceptability construct and weighted response options.**

|  | | | | | | |
| --- | --- | --- | --- | --- | --- | --- |
|  |  | Very Easy | Easy | Neither easy nor difficult | Difficult | Very Difficult |
| Ease of use | | | | | | |
| Ac1 | HCV self-testing is | 5 | 4 | 3 | 2 | 1 |
| Ac2 | HCVST sample is to  collect | 5 | 4 | 3 | 2 | 1 |
| Ac4 | It is to use the HCVST  self-test | 5 | 4 | 3 | 2 | 1 |
| Ac5 | The Instructions were to  follow | 5 | 4 | 3 | 2 | 1 |
| Ac6 | HCVST result is to interpret | 5 | 4 | 3 | 2 | 1 |
| Ac7 | It was to report my self test results to the provider | 5 | 4 | 3 | 2 | 1 |
| Confidence and confidentiality | | | | | | |
|  |  | Strongly Agree | Agree | Neutral | Disagree | Strongly Disagree |
| Ac8 | HCV self-testing is private and  confidential | 5 | 4 | 3 | 2 | 1 |
| Ac9 | I am confident the HCVST results are accurate | 5 | 4 | 3 | 2 | 1 |
| Ac10 | I prefer to self test than have a provider administer a test | 5 | 4 | 3 | 2 | 1 |
| Self-empowerment – feeling able | | | | | | |
| Ac11 | I feel that I can self test  according to the instructions | 5 | 4 | 3 | 2 | 1 |
| Ac12 | I feel that I can understand safe use and can comply, and knows when to seek  assistance | 5 | 4 | 3 | 2 | 1 |
| Ac13 | I feel that I can access information about self testing  and express needs | 5 | 4 | 3 | 2 | 1 |
| Ac14 | I feel that I can exercise choice over whether or not to choose  a self test | 5 | 4 | 3 | 2 | 1 |
| Ac15 | I feel that I can accurately determine when additional care is needed, know who and how to access support, and have ability to access that  follow-up when needed | 5 | 4 | 3 | 2 | 1 |
| Future use | | | | | | |
| Ac16 | I will recommend HCV self-  test to my family and friends | 5 | 4 | 3 | 2 | 1 |
| Ac17 | I will take the HCVST kits home for my family and  friends | 5 | 4 | 3 | 2 | 1 |
| Ac18 | I would use the HCVST again (if needed) | 5 | 4 | 3 | 2 | 1 |

1. **Descriptive statistics; Mean and Standard Deviation**

Table S4.2 summarises item-level descriptive statistics for all 17 indicators. Although Likert items are ordinal values, because responses were highly skewed toward the most favourable category this resulted in medians at or near the upper bound. Therefore means and standard deviations are presented to allow finer discrimination between items^[[1]](#footnote-1)^. Mean scores were uniformly high, indicating strong overall endorsement of positive acceptability statements.

**Table S4.2. Descriptive statistics for indicators**

|  | | | |
| --- | --- | --- | --- |
|  |  | Mean | Standard deviation |
| Ease of use | | | |
| Ac1 | HCV self-testing is | 4.61 | 0.59 |
| Ac2 | HCVST sample is to collect | 4.56 | 0.63 |
| Ac4 | It is to use the HCVST | 4.62 | 0.57 |
| Ac5 | The Instructions were to follow | 4.61 | 0.59 |
| Ac6 | HCVST result is to interpret | 4.67 | 0.53 |
| Ac7 | It was to report my self-test results to the provider | 4.60 | 0.57 |
| Confidence and confidentiality | | | |
| Ac8 | HCV self-testing is private and confidential | 4.65 | 0.54 |
| Ac9 | I am confident the HCVST results are accurate | 4.66 | 0.56 |
| Ac10 | I prefer to self-test than have a provider administer a test | 4.44 | 0.85 |
| Self-empowerment – feeling able | | | |
| Ac11 | I feel that I can self-test according to the instructions | 4.58 | 0.64 |
| Ac12 | I feel that I can understand safe use and can comply, and knows when to seek assistance | 4.54 | 0.67 |
| Ac13 | I feel that I can access information about self-testing and express needs | 4.53 | 0.70 |
| Ac14 | I feel that I can exercise choice over whether or not to choose a self-test | 4.64 | 0.57 |
| Ac15 | I feel that I can accurately determine when additional care is needed, know who and how to access support, and have ability to access that follow-up when needed | 4.50 | 0.69 |
| Future use | | | |
| Ac16 | I will recommend HCV self-test to my family and friends | 4.72 | 0.58 |
| Ac17 | I will take the HCVST kits home for my family and friends | 4.68 | 0.64 |
| Ac18 | I would use the HCVST again (if needed) | 4.76 | 0.45 |

1. **Check data completeness**

Table S4.3 details completeness of item-level data prior to factor analysis. Missingness was minimal across all indicators (< 3.5%), supporting the decision to use complete-case analysis (final N = 1,848). The small proportion of missing responses and absence of systematic patterns reduced the likelihood of bias from listwise deletion.

**Table S4.3. Missingness across response items.**

| Variable | Variable explainer | Missing_Count | Missing_Percent |
| --- | --- | --- | --- |
| ac1 | Overall ease | 6 | 0.3 |
| ac2 | Sample collection | 41 | 2.1 |
| ac4 | Use | 2 | 0.1 |
| ac5 | Following instructions | 4 | 0.2 |
| ac6 | Interpreting results | 9 | 0.5 |
| ac7 | Reporting results | 67 | 3.4 |
| ac8 | Private and confidential | 0 | 0 |
| ac9 | Confidence in result accuracy | 2 | 0.1 |
| ac10 | Preference for self-testing | 6 | 0.3 |
| ac11 | Feel able to self-test | 7 | 0.4 |
| ac12 | Understand safe use | 2 | 0.1 |
| ac13 | Can access info and express needs | 3 | 0.2 |
| ac14 | Exercise choice | 11 | 0.6 |
| ac15 | Understand care needs | 56 | 2.8 |
| ac16 | Recommend to family and friends | 4 | 0.2 |
| ac17 | Take home to family and friends | 4 | 0.2 |
| ac18 | Use the kit again | 4 | 0.2 |

1. **Domain specification, internal consistency checks and factorability**

Table S4.4 presents results of internal consistency and sampling adequacy tests. The overall Cronbach’s α of 0.91 demonstrates excellent internal reliability of the full 17-item pool. The Kaiser–Meyer–Olkin (KMO) value of 0.92 and Bartlett’s Test of Sphericity (p < 0.001) confirm that inter-item correlations were sufficient to justify factor extraction, indicating the data were well suited to exploratory factor analysis^[[2]](#footnote-2),^^[[3]](#footnote-3),^^[[4]](#footnote-4)^.

**Table S4.4. Internal consistency and sampling adequacy tests.**

| **Test type** | **Test score** |
| --- | --- |
| Full scale (Alpha) | 0.91 |
| KMO Value | 0.92 |
| Bartlett’s Test of Sphericity. | <0.001 |

1. **Exploratory Factor Analysis (EFA)**

An exploratory factor analysis was performed using principal-axis factoring with oblimin rotation on the polychoric correlation matrix. Factor retention followed the Kaiser–Guttman criterion (eigenvalues > 1), visual inspection of the scree plot, and results of a 50-iteration parallel analysis^[[5]](#footnote-5)^^[[6]](#footnote-6)^. Three factors exceeded eigenvalues > 1 (9.3, 2.0, 1.5), and both the scree plot and parallel analysis supported a three-factor solution (**Figure S4.1**).

**Figure S4.1. Scree plot and parallel analysis supporting a three-factor solution**


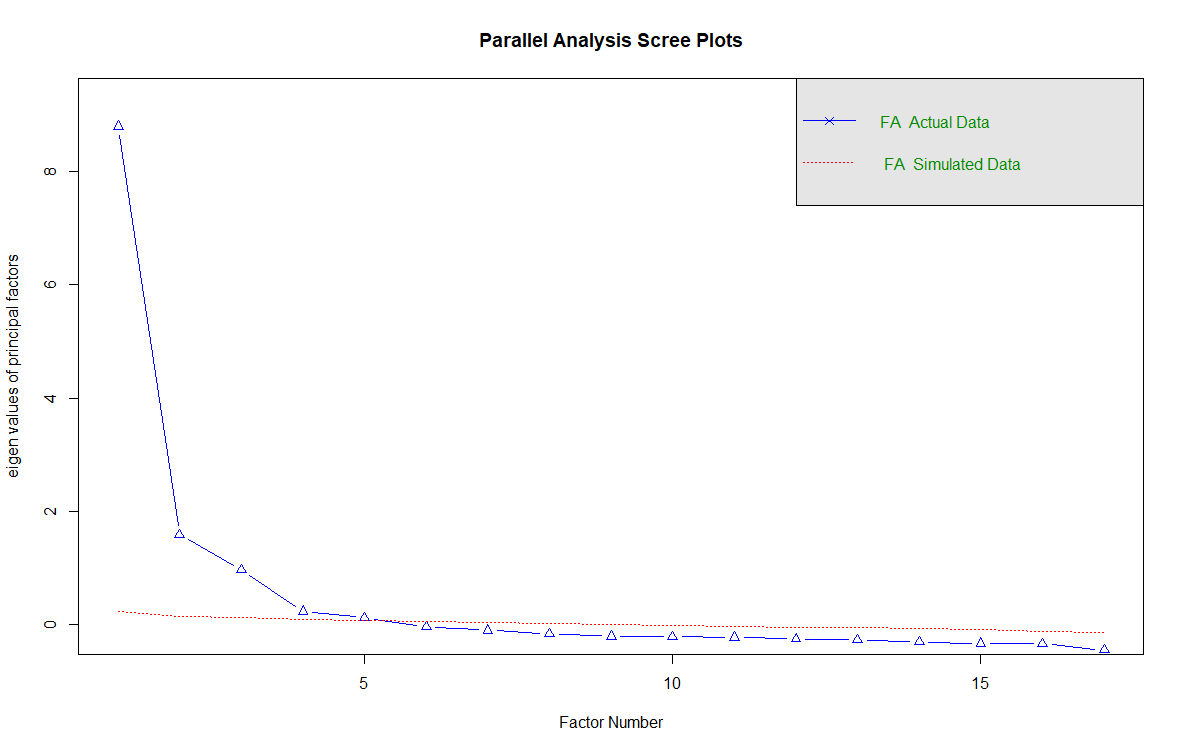


Table S4.5 displays the pattern matrix of factor loadings and communalities. The EFA identified three interpretable domains: Empowerment, Ease of Use, and Future Use Intentions that together explained the majority of common variance.

**Table S4.5. Pattern matrix of factor loadings and communalities.**

| Item | Variable explainer | Self-empowerment | Ease of use | Future use | Communality |
| --- | --- | --- | --- | --- | --- |
| ac1 | Overall ease | -0.05 | 0.926 | -0.019 | 0.789 |
| ac2 | Sample collection | -0.053 | 0.902 | -0.019 | 0.745 |
| ac4 | Use | 0.012 | 0.925 | -0.041 | 0.833 |
| ac5 | Following instructions | 0.111 | 0.812 | -0.013 | 0.767 |
| ac6 | Interpreting results | 0.014 | 0.81 | 0.06 | 0.722 |
| ac7 | Reporting results | 0.041 | 0.732 | 0.126 | 0.684 |
| ac8 | Private and confidential | 0.137 | 0.245 | 0.381 | 0.416 |
| ac9 | Confidence in result accuracy | 0.142 | 0.159 | 0.452 | 0.422 |
| ac10 | Preference for self-testing | 0.328 | 0.107 | 0.335 | 0.437 |
| ac11 | Feel able to self-test | 0.87 | 0.034 | -0.01 | 0.783 |
| ac12 | Understand safe use | 0.948 | -0.003 | -0.017 | 0.877 |
| ac13 | Can access info and express needs | 0.93 | 0.026 | -0.019 | 0.873 |
| ac14 | Exercise choice | 0.793 | 0.033 | 0.03 | 0.691 |
| ac15 | Understand care needs | 0.907 | -0.069 | 0.032 | 0.785 |
| ac16 | Recommend to family and friends | -0.039 | -0.011 | 0.94 | 0.833 |
| ac17 | Take home to family and friends | 0.008 | -0.033 | 0.882 | 0.76 |
| ac18 | Use the kit again | 0.041 | 0.049 | 0.728 | 0.606 |

Items ac8–ac10 from the original “Confidence and Confidentiality” domain showed weak loadings (< 0.45) and low communalities (< 0.45) and were therefore excluded from confirmatory analyses. The final three-factor solution showed conceptual coherence and simple structure, providing the basis for the CFA model.

1. **Confirmatory factor analysis**

To validate the factor structure derived from the EFA, a three-factor confirmatory factor analysis was estimated using the WLSMV estimator for ordered categorical data. Model fit indices indicated good overall fit (CFI = 0.996; TLI = 0.995; RMSEA = 0.060; SRMR = 0.042). All items loaded significantly (p < 0.001) on their respective latent factors with standardised loadings from 0.81 to 0.95, and moderate inter-factor correlations (0.49–0.60). These findings support the robustness and discriminant validity of the three identified domains.

1. **Second order CFA**

To examine whether the three first-order domains could be represented by a single higher-order construct, a second-order CFA was specified in which Ease of Use, Empowerment, and Future Use Intentions loaded onto an overarching latent factor of Acceptability. Model fit indices were identical to those of the three-factor model (CFI = 0.996; TLI = 0.995; RMSEA = 0.060; SRMR = 0.042), consistent with theoretical equivalence between the two structures^[[7]](#footnote-7)^. The second-order factor loadings (Empowerment: 0.84, Ease of Use: 0.71 and Future Use Intentions: 0.68) demonstrate that the higher-order latent construct adequately explains shared variance across domains, justifying the derivation of a unified acceptability score for subsequent analyses.

1. **Refined acceptability scores**

Refined acceptability scores were generated for each participant using least squares regression method. Figure S4.2 illustrates the distribution of the continuous acceptability scores derived from the second-order CFA. The distribution was left-skewed, indicating high overall acceptability of HCV self-testing within the sample.

**Figure S4.2. Histogram of refined acceptability score distribution**


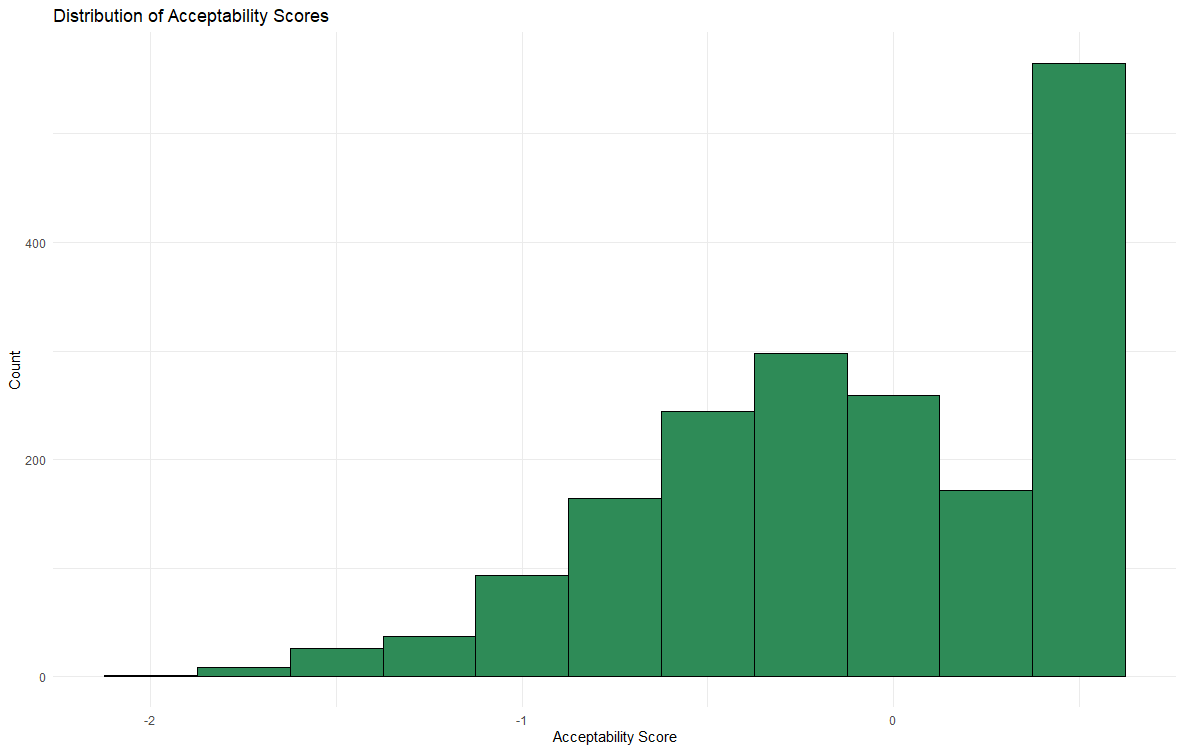


This supports the interpretation that HCV self-testing was highly acceptable in this sample, with only a small minority indicating lower acceptability across empowerment, ease of use, and future use intentions.

1. **R Session Info / Packages Used**

All analyses were conducted using R version 4.3.2, with key packages including tidyverse for data management, psych for exploratory factor analysis, and lavaan for confirmatory factor analysis and structural equation modelling. The GPArotation package was used to enable oblique factor rotation.

1. Norman, G. Likert scales, levels of measurement and the “laws” of statistics. Adv Health Sci Educ Theory Pract. 2010 Dec;15(5):625-32. [↑](#footnote-ref-1)
2. Field, A, Miles J, Field Z. Discovering Statistics Using R. London: SAGE Publications Ltd; 2012. [↑](#footnote-ref-2)
3. Fabrigar LR, Wegener DT. Exploratory Factor Analysis. Online edn, Oxford Academic; 2011. <https://doi.org/10.1093/acprof:osobl/9780199734177.001.0001>, accessed 7 Oct 2025. [↑](#footnote-ref-3)
4. Kaiser HF (1974). An index of factorial simplicity. Psychometrika, 1974 Mar;39(1):31-36. [↑](#footnote-ref-4)
5. Fabrigar L, Wegener DT, MacCallum RC, Strahan EJ. Evaluating the use of exploratory factor analysis in psychological research. Psych Meth. 1999 Sep;4(3): 272-299. [↑](#footnote-ref-5)
6. Zwick WR, Velicer WF. (1986). Comparison of five rules for determining the number of components to retain. Psych Bull. 1986 May;99(3):432-442. [↑](#footnote-ref-6)
7. Brown TA. Confirmatory Factor Analysis for Applied Research. 2nd ed. New York: Guilford Press; 2015. [↑](#footnote-ref-7)
