# Supplementary material for: Feasibility and acceptability of hepatitis C virus self-testing models among high-risk groups in Nasarawa, Nigeria; Exploratory cross-sectional analysis of an implementation study
Source: PLOS Glob Public Health. 2026 Jun 29;6(6):e0005567. doi: 10.1371/journal.pgph.0005567 (PMC13313356; doi:10.1371/journal.pgph.0005567)
Supplement: S1 Checklist — (DOCX) [file pgph.0005567.s005.docx]

Inclusivity in global research

PLOS’ policy on inclusivity in global research aims to improve transparency in the reporting of research performed outside of researchers’ own country or community and ensures that PLOS publications reporting global research adhere to high standards for research ethics and authorship. Authors of relevant research articles may be asked to complete the questionnaire below, which outlines ethical, cultural, and scientific considerations specific to inclusivity in global research. This questionnaire may be requested when researchers have travelled to a different country to conduct research, if research uses samples collected in another country, research with Indigenous populations or their lands, or if research is on cultural artefacts. Researchers travelling to another country solely to use laboratory equipment will not normally be required to complete the questionnaire. However, the questionnaire can be requested at the journal’s discretion for any submission – if you have been requested to complete this questionnaire by the PLOS journal you submitted to, please do so.

Please complete the questionnaire below and include this as a Supporting Information file with your manuscript. Note that if your paper is accepted for publication, this checklist will be published with your article in the supporting information files. Please ensure that you reference the checklist in the main body of your manuscript. We suggest adding a subsection ‘Inclusivity in global research’ to your Methods section and adding the following sentence: “Additional information regarding the ethical, cultural, and scientific considerations specific to inclusivity in global research is included in the Supporting Information (SX Checklist)”

The questions have been designed to be applicable to a wide range of study types, and there are subsections for both human subjects research and non-human subjects research. If any of the questions are not relevant to your research please mark them as “N/A” as appropriate.

**Ethical considerations, permits and authorship**

*This section is applicable to all research types.*

Provide details as to who granted permissions and/or consent for the study to take place in the Methods section of your manuscript. This should include the names of **all** ethics boards, governmental organizations, community leaders or other bodies that provided approval for the study. If individuals provided approval refer to these people by their role or title but do not list their name(s).

Ethical approval for the study was obtained from the John Hopkins University Bloomberg School of Public Health Institutional Review Board (BPSH #20755), National Health Research and Ethics Committee in Nigeria (approval number NHREC/01/01/2007-17/07/2022), Nasarawa State Ministry of Health Ethics Review Committee (approval number NHREC 18/06/2017), and World Health Organization Ethics Review Committee (WHO ERC 3809). Study participants provided written informed consent.

If there were any deviations from the study protocol after approval was obtained please provide details of these changes in the Methods section of your manuscript.

Reported on page number: N/A *(No protocol deviation recorded during the study implementation)*

Did this study involve local collaborators that are residents of the country where the research was conducted or members of the community studied? If you do not have any authors from said communities, please provide an explanation for this below.

*The project team held a series of consultative discussions with a wide range of national and state stakeholders including civil society organizations and key populations networks. Relevant stakeholders, including government ministries, departments and agencies (MDAs), healthcare workers and community members were adequately sensitized about the study and the overall HCVST project in Nigeria, thus ensuring adequate buy-in of the project by the stakeholders.*

*The project team also engaged with a wide range of national and state stakeholders including CSOs and key populations networks in the implementation of the HCVST study. The CSOs were involved in the design, implementation and monitoring of research activities and outcomes, including developing a joint plan to guide the HCVST study implementation. They played a critical role in creating awareness about the study, mobilizing communities, and providing useful advice and feedback to the research team as members of the community advisory board (CAB). The CSOs engaged include the Family Health Care Foundation (FAHCI),*

*Beacon Youth Initiative (BYI), Child Education and Community Development Initiative (CECDI)*

*and AL-MIRSAN Nutrition and Health Foundation.*

*A few government stakeholders are authors on this manuscript.*

Everyone listed as an author should meet PLOS’ criteria for authorship and all individuals who meet these criteria should be included in the author byline, rather than the acknowledgements. For further information please see the journal’s Authorship Policy.

**Human subjects research (e.g. health research, medical research, cross-cultural psychology)**

Did you obtain written informed consent from a representative of the local community or region before the research took place? How did you establish who speaks for the community? Details of written informed consent obtained from study participants should be reported separately in the Methods section of your manuscript.

*Yes, we obtained ethical approval from the Nasarawa State Ministry of Health for this study.*

How did members of the local community provide input on the aims of the research investigation, its methodology, and its anticipated outcome(s)?

*The project team held a series of consultative discussions with a wide*

*range of national and state stakeholders including civil society organizations and key*

*populations networks. Relevant stakeholders, including government ministries, departments and*

*agencies (MDAs), healthcare workers and community members were adequately sensitized*

*about the study and the overall HCVST project in Nigeria, thus ensuring adequate buy-in of the*

*project by the stakeholders. In addition the study team convened a community advisory board and held the monthly CAB meetings provided updates on HCVST study to relevant stakeholders, get feedback and recommendations that could improve the study's quality. These updates were reviewed, and constructive feedback provided by board members towards the success of the study. For instance, in response to some of the CAB feedback, the study team developed HCVST waste management standard operating*

*procedure (SOP) to guide the study in the correct disposal of HCVST test kits to safeguard*

*against spread of infection.*

When engaging with the local community, how did you ensure that the informed consent documents and other materials could be understood by local stakeholders?

*All information sheets and consent forms were translated into Hausa. Trained research assistants, fluent in English and Hausa obtained consent from potential participants and verbally explained the study to them, providing all relevant information (purpose, procedures, risks, benefits, alternatives to participation, etc.) using the appropriate information sheet for each use case. Providers at each study site introduced the study to their clients and if the clients were interested in learning more about the study, they were referred to the Research Assistants who were located nearby. Potential participants were given ample opportunity to ask questions, and all their queries were answered. Each participant was given a written information sheet to read. If they couldn't read, the research assistant read the consent form to them in the language they understood. Potential participants were given time to reflect on their participation in the study, and their understanding of the study was assessed by asking them to describe the purpose of the study and what was expected of participants.*

Will the findings of the research be made available in an understandable format to stakeholders in the community where the study was conducted (e.g. via a presentation, summary report, copies of publications, etc.)? Please provide details of how this will be achieved.

*Yes, the project leveraged various platforms, including state, national, regional, and*

*international fora to disseminate its achievements and findings from the HCVST exploratory*

*study. These include the community advisory board (CAB), national harm reduction TWG (2nd7th July 2023), National Viral Hepatitis TWG, Nasarawa State Viral Hepatitis TWG, African Viral*

*Hepatitis Summit (October 23-26, 2023), International AIDS Conference in Brisbane Australia*

*(23-26 July 2023), 22nd edition of the International Conference on AIDS & STIs in Africa*

*(ICASA 2023) held in Zimbabwe from 4th – 9th December 2023, and World Hepatitis Summit*

*held in Portugal from April 9-11, 2024. The team provided technical support to*

*stakeholders, ensuring that the findings from the HCVST study strategically inform and enhance*

*national and subnational viral hepatitis programs.*

**Non-human subjects research using specimens/ animals collected as part of the study, or those housed in archival collections. Examples include archaeology, paleontology, botany and zoology.**

Did the permission you obtained from a local authority to perform the study include an agreement on access to outputs and benefit sharing? This may include procedures to enable fair distribution of the benefits and resources arising from the research performed. Please include any details of Prior Informed Consent and Benefit Sharing Agreements obtained. These may be required by field-specific regulations, for example the Convention on Biological Diversity (CBD) and the associated Nagoya Protocol.

*No specimens were archived from the study. All materials used for testrning were discarded after the test.*

If the material used in your study was imported, please A) provide the year it was imported and B) indicate whether permits were obtained to import/export the materials used, C) provide details of any permits obtained. If this information is not available, please indicate this.

*The rapid diagnostic test kits were imported in 2023. STAR Nigeria collaborated with the National Agency for Control of AIDS and National Agency for Food and Drug Administration and Control in obtaining an import duty exemption certificate (IDEC) waiver for oral and blood based HCVST kits. The project team also received a waiver to use oral and blood based HCVST in ‘research context only’ from Medical Laboratory Science Council of Nigeria (MLSCN).*

If you used archival specimens, please state how the material used in your study was acquired by the institute it is held in and provide details of any permits obtained for the original excavations/ sample collection. If this information is not available, please indicate this.

*N/A*

How was the potential cultural significance of the materials collected in your study to local communities considered in your research design? Were Indigenous peoples and/or local researchers and institutions involved with archaeological excavations / collection of specimens? If so, please provide a description of their involvement.

*The study did not involve the collection of culturally significant materials, archaeological excavations, or biological specimens.*

If your manuscript includes photographs of human remains please indicate whether authors obtained permission from descendants or affiliated cultural communities to do so.

*N/A*
