# Supplementary material for: Feasibility and acceptability of hepatitis C virus self-testing models among high-risk groups in Nasarawa, Nigeria; Exploratory cross-sectional analysis of an implementation study
Source: PLOS Glob Public Health. 2026 Jun 29;6(6):e0005567. doi: 10.1371/journal.pgph.0005567 (PMC13313356; doi:10.1371/journal.pgph.0005567)
Supplement: Attachment — Submitted filename: Cover_letter_reviewer_response_R1.doc [file pgph.0005567.s008.doc]

**Response to reviewers**

**PLOS Global Public Health**

22 January 2026.

Dear Dr Orvalho Augusto,

**Re: PGPH-D-25-03540; Feasibility and acceptability of hepatitis C virus self-testing models among high-risk groups in Nasarawa, Nigeria; exploratory cross-sectional analysis of an implementation study.**

Thank you for facilitating the review of the above titled manuscript. Please see below a point-by-point response to each reviewers’ comments.

Please note that all line numbers refer to clean version of this manuscript.

| **No.** | **Comment (Editor and Reviewer)** | **Author Response / Change Made** | **Location in Revised Manuscript (Line refers to track changes version)** |
| --- | --- | --- | --- |
| **Response to journal requirements** | | | |
| 1 | Please include a complete copy of PLOS’ questionnaire on inclusivity in global research in your revised manuscript. | Thank you, this has been re-uploaded as a Supporting Information file (S5) and listed in the supporting documents in the manuscript. | Supporting Information (S5 File) |
| 2 | Please amend your detailed Financial Disclosure statement. This is published with the article. It must therefore be completed in full sentences and contain the exact wording you wish to be published. | Thank you, we have amended our detailed financial disclosure statement on the online platform to the following:  *“This work was supported by Unitaid grant (2017-16-PSI-STAR to KH). The funders had no role in study design, data collection and analysis, decision to publish, or preparation of the manuscript.”* | Online disclosures. |
| 3 | Please clarify all sources of funding (financial or material support) for your study. List the grants (with grant number) or organizations (with url) that supported your study, including funding received from your institution. |  | Online disclosures |
| 4 | State the initials, alongside each funding source, of each author to receive each grant. |  | Online disclosures. |
| 5 | State what role the funders took in the study. If the funders had no role in your study, please state: “The funders had no role in study design, data collection and analysis, decision to publish, or preparation of the manuscript.” |  | Online disclosures. |
| 6 | If any authors received a salary from any of your funders, please state which authors and which funders. |  | Online disclosures. |
| 7 | We do not publish any copyright or trademark symbols that usually accompany proprietary names, eg (R), (C), or TM. Please remove all instances of trademark/copyright symbols throughout the text, including ® on page 7. | Thank you, we have removed these symbols. | Lines 173, 175. |
| 8 | Please indicate how the 2000 sample size was determined. | Thank you, we have added in a sample size section.  “*The sample size was determined by implementation research objectives and pragmatic recruitment considerations. A sample of 2000 participants permits estimation of an identified prevalence of 11.4% with a precision of approximately ±1.4 percentage points at the 95% confidence level.*” | Lines 138-141 |
| 9 | Line 132 is a bit unclear. Can it be rewritten? | Thank you, we have rewritten line 132 to clearly state that participants received health information on HCV as well as the study procedures:  *“Once enrolled, study participants received health information on HCV transmission, prevention, and treatment availability, as well as instructions on how to conduct either finger-prick blood-based or oral fluid-based HCV antibody self-test, interpret the results and linkage procedures in the case of a reactive test.”* | Line 146-9 |
| 10 | About the acceptability, it needs to be recognized that this instrument has never been tested in this population before. | Thank you for raising this important point; we have included a sentence in the methods that this instrument has never been tested in this population before.  *“The survey was generated de novo for this study and had not been tested prior in this context.”* | Line 254-5 |
| 11 | The EFA and CFA results are good, but it is not a guarantee that this will work forever. | This is very true. We touch on the importance of not overstating these findings in the discussion:  “*Additionally, we did not assess whether the acceptability scale functioned equivalently across ART and OSS settings, so observed differences should be interpreted with that caveat; no assurances can be made that this scale will function equivalently in different contexts.”* | Lines 545-73. |
| 12 | For the study of distribution, as in figure 3, I would suggest a cumulative counts plot. | Thank you, we have amended and re-uploaded this figure, as well as the supporting text. | Line 313, 406, 412.  Figure 3 |
| 13 | About the “least 10% of acceptability” concept. Can we call this the lowest decile acceptability score? | This is a far better way of framing this, and we have implemented this throughout the manuscript. | Lines 38, 283, 312, 317, 407, 413, 415, 425, 429, 435, 438. |
| 14 | Figure 4… fails to tell us the proportion of observations below the line of the overall lowest decile. I suggest adding a table with such proportions. | Thank you for this comment, we have now added integers under the figure that shows the proportions in the lowest decile. | Figure 4 |
| 15 | Figure 2 is a bit deceptive… compute accurate proportions (in percentages) and add 95% confidence intervals for the cascade plot. | Thank you – we have revised this figure to show the proportions, with binomial exact confidence intervals, with absolute numbers reported underneath the cascade. We have updated the figure title. | Figure 2 |
| **Reviewer #1.** | | | |
| 16 | Has the statistical analysis been performed appropriately and rigorously? Reviewer #1: No | Thank you for your response to this question; we hope that the responses below alleviate your concerns as to the appropriateness and rigour of our statistical analysis. | - |
| 17 | Have the authors made all data underlying the findings in their manuscript fully available? Reviewer #1: No | The data was included as a supplement with the manuscript – we have reuploaded this to ensure it is explicitly titled (S6_data) for your review. | - |
| 18 | Clarify that use of HCVST is referring only to antibody testing (this applies to the paper throughout). | Thank you, we have explicitly clarified in the abstract, and methods that HCV self-testing in this study refers to HCV antibody self-testing, with reactive results requiring confirmatory HCV RNA testing prior to treatment:  *Abstract:*  *“Participants tested with either blood-based or oral fluid antibody HCVST, and with or without health worker support. Follow-up documented results and linkage to qualitative RNA PCR confirmatory testing and treatment.”*  *Methods*  *“The self-tests used in this study detect HCV antibodies (not viraemia) and therefore required confirmatory HCV RNA testing to diagnose current infection and determine treatment eligibility.”* | Lines 33-6  Lines 187-8. |
| 19 | - The selection criteria is difficult to follow and should be clarified - perhaps with dot points? | Thank you for your comment, we have bullet-pointed the criteria for participant selection in the methods:  *“The study enrolled 2,000 clients aged ≥18 years. Inclusion criteria were:*   - *Living with HIV and accessing care at participating ART clinics or,* - *Seeking services at participating OSS, and* - *with a negative or unknown HCV status at enrolment.”* | Lines 131-4 |
| 20 | - Specify that "blood-based" testing means finger prick? | Thank you, we have made sure to reference finger-prick whenever we mention blood-based. | Lines 34, 103-4, 147, 172, 177, 210, 310. |
| 21 | - Again, need to clarify that testing is antibody, correct? Presuming also that RA information discussed testing procedures – i.e. what happened if the ST was positive and it’s interpretation? | Thank you, we have addressed ensuring that testing is antibody testing (see comment 18).  You are correct, that the RA also discussed testing procedures, i.e., what happened if the test was positive – we have expanded the implementation detail:  *“Clients interested in learning more about the study were introduced to a trained research assistant (RA) who described the study in detail. Once enrolled, study participants received health information on HCV transmission, prevention, and treatment availability, as well as instructions on how to conduct either finger-prick blood-based or oral fluid-based HCV antibody self-test, interpret the results and linkage procedures in the case of a reactive test.”* | Lines 144-9. |
| 22 | - How many of each test did you have? Why did you not have enough tests so that any participant could choose whichever test they wanted for the entirety of the study period? | We procured relatively equally numbers of test kits (1030 finger-prick blood tests and 965 oral fluid tests), assuming sites would access relatively equal numbers of tests (up to 500 tests total per site). However, given the preference for finger-prick blood tests in ART sites, and later recruitment in OSS sites, we depleted our stock of finger-prick blood-based tests before we finished recruitment in OSS sites.  We have included the following additional sentence to clarify this in the methods:  *“Because research-use supplies were finite, uptake differed by site and recruitment happened later in OSS sites than ART sites, finger-prick blood-based kits were depleted earlier through use in ART sites than oral-fluid kits. As a result, OSS participants enrolled after 13 November 2023 could only receive oral fluid–based HCVST”* | Lines 176-9. |
| 23 | - Presuming drug abstention is not a requirement to treatment initiation? | Thank you, we have now added this information under implementation procedure section on treatment that *“Drug abstention was not a prerequisite for confirmatory testing or treatment initiation, and treatment eligibility followed the national clinical pathway for HCV RNA-confirmed infection.”* | Lines 196-8 |
| 24 | - You explain later in the paper that participants could be part of multiple KP categories - but probably good to explain in the "exposure variables" section. | We have added this the exposure variable section: *“Participants could report more than one key population identity.*” | Lines 227-8 |
| 25 | I would clarify that analysis is only based on 1,995 valid participants. | Thank you, we have revised the abstract and methods accordingly to reflect case-complete feasibility and acceptability analyses.  *Abstract:*  *Case-complete feasibility analyses were conducted across the HCV care cascade using chi-square tests (N=1995). Acceptability was evaluated using a post-test survey score derived through case-complete factor analysis (N=1868).*  *Methods:*  *“Case-complete feasibility analysis (N=1995) was conducted. Only five participants were excluded from analysis as they were missing demographic and outcome data.”* | Lines 36-8.  Lines 235-6. |
| 26 | Perhaps just a quick sentence describing the nature of Nigeria's epidemic - i.e. is it generalised or population specific? | Thank you, we have clarified that:  *“the Nigerian epidemic is generalized with disproportionate burden among key populations.”* | Lines 84-5 |
| 27 | Needs to be more information about participant recruitment… no information about the refusal rate. | We have clarified that refusal rates were not systematically recorded, and that enrolment reflects uptake among eligible clients who consented rather than a population-level acceptance rate of HCVST:  *“The study did not systematically record the number or characteristics of clients who declined participation after receiving study information. As such, enrolment counts reflect uptake among eligible clients who consented to participate, rather than a population-level acceptance rate of HCV self-testing.”* | Lines 243-6. |
| 28 | Very little in methods about your qualitative interviewing - this needs expansion. | Thank you – we have included additional detail on the qualitative component - this was not interviewing, but thematic analyses of free-text response from participants who completed the endline survey (S2) specifically in response to questions 9, 40, 41, 42 with non-missing N=1980 responses.  “*Qualitative exploration*  *Qualitative thematic analysis of free-text responses to four open-ended questions in the endline survey (S2) was conducted to provide additional insights and enrich quantitative cascade differences and acceptability. Free-text response questions elicited clients’ perspectives on preferences in terms of test-type, service location, and assistance modality as well as previous experiences with self-test technologies. A total of N=1980 non-missing responses to at least one free-text item were recorded.*  *Responses were analysed using an exploratory, inductive thematic approach. Two members of the study team independently reviewed responses to develop an initial coding framework (MS and YD), which was iteratively refined through discussion. Coding focused on identifying recurring themes and contrasts by site type (ART clinic vs OSS) to contextualise observed differences in acceptability and cascade outcomes. Qualitative findings were used to enrich interpretation of quantitative results rather than to generate standalone explanatory theory.”*  We have also clarified the role of qualitative findings to synthesize the quantitative data in the statistical analysis section:  *“Qualitative themes were examined by site type (ART clinic vs. OSS) and integrated with quantitative findings to contextualise observed differences in cascade outcomes and acceptability.”* | Lines 285-299  Lines 319-21. |
| 29 | Very little satisfactory information about the acceptability questionnaire… factor analysis. | We appreciate the reviewer’s comments that there is little satisfactory information about the factor analysis in the main manuscript; we had attempted to balance the methodological data between the main manuscript and the supplementary material (**S4**) in the interests of journal space requirements; below we address the statistical questions that we hope may resolve this.  We also look forward to hearing any additional comments on how we could further strengthen the main manuscript by including additional detail. | - |
| 30 | How did you determine that 10% equals 'unacceptable'? | This is an important point; in the results we describe the generally high acceptability scores after visual inspection of the histogram (**S4**). NB. We have now amendeded this to a cumulative counts plot as per response to comment 12.  The “lowest 10% of acceptability was chosen” as the cut-off, both to reflect the minority of respondents within this highly left-skewed data, while balancing the power requirements (n=186) for regression comparisons.  We have updated the methods to clarify this:  *“Given generally high rates of acceptability and left skew data distribution, and the need to characterise the minority of respondents with comparatively lower acceptability while retaining sufficient sample size for regression analyses, the continuous score was split into a binary outcome between the lowest decile acceptability score and the remaining acceptability scores for statistical analysis (****S4****).”*  However, in response to comment 13 above, we also believe that the use of term “unacceptable” is likely far too loose a framing, given that overall, the distribution of acceptability scores was very high, and so we have changed the narrative throughout to “*lowest decile of acceptability*”. This therefore does not overstate the lowest decile as “unacceptable.” | Lines 280-2 |
| 31 | Non-considering possible refusal rates… essentially 100% completed the self-test? | In the feasibility outcome description, we have clarified that refusal rates were not collected (comment 27).  We have also included this as a limitation in the discussion:  *“Moreover, acceptability findings in this study reflect perceptions and experiences among participants who consented to HCVST and completed post-test assessments. Because refusal rates were not systematically captured, we cannot estimate the proportion of all eligible clients who declined participation. Therefore, high enrolment and acceptability scores should not be interpreted as indicating universal acceptability of HCVST among all clinic or OSS attendees, but rather as evidence that HCVST is highly acceptable among those willing to engage with self-testing when offered.”* | Lines 533-39. |
| 32 | Why wasn't demographic information collected at baseline? | Demographic information was collected at baseline:  *“Demographic, behavioral and clinical characteristics of study participants, as well as HCVST type used (finger-prick blood-based or oral fluid), approach (observed or unobserved), and facility type (ART clinic or OSS) were collected on an intake form administered upon study enrollment (S1) and from an endline survey completed post-test (S2).”*  There were five participants who’s baseline demographic data and outcome data was missing, who we consequently excluded from the analysis – we have added in explanation on this under the feasibility outcome (comment 25), as well as cleaned up the results section were this data was erroneously presented:  *“Case-complete feasibility analysis (N=1995) was conducted. Only five participants were excluded from analysis as they were missing demographic and outcome data.”* | Lines 209-12  Lines 235-36. |
| 33 | Suggest re-doing Figure 2… Add percentages to columns. | Thank you, we have redone the figure accordingly. | Figure 2 |
| 34 | OSS clients are much younger and more males… how much did this impact outcomes? | We have added a sentence exploring how the demographic structure of OSS clients as more males and younger than ART attendees may have influenced the impact outcomes:  *“Lower acceptability and treatment may also reflect the structural difference in populations by site; OSS clients were generally younger and male compared to the ART sites; these populations are less likely to engage and be retained in care.”* | Lines 503-6. |
| 35 | Did you consider differences across test type? | Yes, we did consider differences in feasibility and acceptability by test type and ran these exploratory analyses prior to compiling our manuscript. However, we felt that because of the stock-outs of finger-prick blood-based tests after 13 November 2023, this limited the interpretability of these results, and these were therefore not presented so as not to obscure the main outcome of the site level differences. | - |
| 36 | I'd include explanation of the SVR12 limitation somewhere higher in the paper. | We have added an explicit statement re- SVR12 results in the methods:  *“SVR12 outcomes were only available for participants who had reached 12 weeks post treatment by the end of data collection, and completed SVR12 testing, as follow up ended before all participants completed 12 weeks.”*  We have also ensured the logic of SVR12 tracks in reporting the results. | Lines 238-39  Lines 382-98. |
| 37 | - The statement about PWID ‘surprise’ may be misplaced. How many PWID did you approach, who refused to participate? How does the number recruited compare against the OSS client list? Also, it’s 28% (PWID) compared to (31% MSM) – are you surprised about MSM? | That is a very important point – we better frame the “surprise” in the following way in the discussion, i.e., not in relation to the proportion of MSM who engaged, but against a backdrop of high HCV burden in this group and the extent of harm reduction activities being offered in OSS:  *“Finally, the proportion of participants identifying as PWID within OSS attendees (28%) was lower than anticipated given their broader HCV burden and the amount of harm reduction programming offered through the OSS.”* | Lines 510-12. |
|  | Additional revisions – not specified by reviewers | While responding to this review, we also made the following changes for clarity:   - Education exposure variable was clarified as “*referring to the highest level of education obtained to date*” - The implications of the study were integrated into the conclusion section of the discussion to better align with conventional reporting guidelines. | Line 218  Lines 582-93. |
