# Supplementary material for: Feasibility and acceptability of hepatitis C virus self-testing models among high-risk groups in Nasarawa, Nigeria; Exploratory cross-sectional analysis of an implementation study
Source: PLOS Glob Public Health. 2026 Jun 29;6(6):e0005567. doi: 10.1371/journal.pgph.0005567 (PMC13313356; doi:10.1371/journal.pgph.0005567)
Supplement: Attachment — Submitted filename: Cover_letter_reviewer_response_R2_090426.docx [file pgph.0005567.s009.docx]

**Response to reviewers**

**PLOS Global Public Health**

10 April 2026.

Dear Dr Johanna Pruller,

**Re: PGPH-D-25-03540R1; Feasibility and acceptability of hepatitis C virus self-testing models among high-risk groups in Nasarawa, Nigeria; exploratory cross-sectional analysis of an implementation study.**

Thank you for facilitating the review of the above titled manuscript. Please see below a point-by-point response to each reviewers’ comments.

Please note that all line numbers refer to tracked changes version of this manuscript.

| **No.** | **Comment (Editor and Reviewer)** | **Author Response / Change Made** | **Location in Revised Manuscript** |
| --- | --- | --- | --- |
| **Response to Reviewer 1** | | | |
| 1 | Given the noted limitation of not being able to demonstrate comprehensive acceptability (without refusal rates) – it’s advised to further review the paper for instances of potential overstatement of findings. For example, on page 20. “…while both…facilities demonstrated high engagement…” – 1,000 clients is definitely a lot, but without the context of how many overall clinic clients there are, and how many may have refused, it would be prudent to temper the statements slightly. | Thank you for this comment; we have amended the manuscript accordingly to avoid overstating the level of engagement given the reviewer’s comment; we note that the overstatement stemmed from a loose use of the term “engagement.” We have removed the sentence on page 20. We have also ensured that all interpretations are now explicitly restricted to participants who engaged with HCVST, and avoided assertions of demonstrating acceptability, instead describing acceptability scores given point 2 below. | Lines 51, 398-400, 405, 407-9, 412-13, 414, 498-500. |
| 2 | Importantly, the additional information provided by the authors about their bespoke acceptability measure gives me greater reservations – considering it has no prior validity testing. This is not addressed in the limitations and definitely should be. | Thank you for this important comment – we have incorporated additional detail in the limitations section on the acceptability measure, clarifying that this was a de novo, context-specific measure and that the factor analysis was used to identify latent structure within survey items rather than to develop or validate a formal psychometric instrument. We have also raised this limitation higher in the limitations section so it is not obscured by the other text:  *“Importantly, the acceptability measure used in this study was generated de novo and had not been tested prior in this context. Although there was high internal consistency and the tool demonstrated a coherent factor structure with good model fit under exploratory and confirmatory factor analyses, this approach was intended to identify latent structure within acceptability-related survey items rather than to develop or validate a formal psychometric instrument. It therefore remains plausible that, without prior validation, the constructs identified may not align fully with established theoretical frameworks of acceptability and instead reflect empirical response domains within this implementation context.”* | Lines 473-483. |
