# Supplementary material for: Feasibility and acceptability of hepatitis C virus self-testing models among high-risk groups in Nasarawa, Nigeria; Exploratory cross-sectional analysis of an implementation study
Source: PLOS Glob Public Health. 2026 Jun 29;6(6):e0005567. doi: 10.1371/journal.pgph.0005567 (PMC13313356; doi:10.1371/journal.pgph.0005567)
Supplement: Peer review history

# Peer review: Decision Letter 0

**DOI:** 10.1371/journal.pgph.0005567.r001

**Authors:** Orvalho Augusto

5 Jan 2026

PGPH-D-25-03540

Feasibility and acceptability of hepatitis C virus self-testing models among high-risk groups in Nasarawa, Nigeria; exploratory cross-sectional analysis of an implementation study.

PLOS Global Public Health

Dear Dr. Dunkley,

Thank you for submitting your manuscript to PLOS Global Public Health. After careful consideration, we feel that it has merit but does not fully meet PLOS Global Public Health’s publication criteria as it currently stands. Therefore, we invite you to submit a revised version of the manuscript that addresses the points raised during the review process.

Please submit your revised manuscript by Feb 19 2026 11:59PM. If you will need more time than this to complete your revisions, please reply to this message or contact the journal office at globalpubhealth@plos.org. When you're ready to submit your revision, log on to https://www.editorialmanager.com/pgph/ and select the 'Submissions Needing Revision' folder to locate your manuscript file.

Please include the following items when submitting your revised manuscript:

- A letter that responds to each point raised by the editor and reviewer(s). You should upload this letter as a separate file labeled 'Response to Reviewers'.
- A marked-up copy of your manuscript that highlights changes made to the original version. You should upload this as a separate file labeled 'Revised Manuscript with Track Changes'.
- An unmarked version of your revised paper without tracked changes. You should upload this as a separate file labeled 'Manuscript'.

Guidelines for resubmitting your figure files are available below the reviewer comments at the end of this letter.

We look forward to receiving your revised manuscript.

Kind regards,

Orvalho Augusto, MD, MPH, PhD

Academic Editor

PLOS Global Public Health

Journal Requirements:

1. Please include a complete copy of PLOS’ questionnaire on inclusivity in global research in your revised manuscript. Our policy for research in this area aims to improve transparency in the reporting of research performed outside of researchers’ own country or community. The policy applies to researchers who have travelled to a different country to conduct research, research with Indigenous populations or their lands, and research on cultural artefacts. The questionnaire can also be requested at the journal’s discretion for any other submissions, even if these conditions are not met.  Please find more information on the policy and a link to download a blank copy of the questionnaire here: https://journals.plos.org/globalpublichealth/s/best-practices-in-research-reporting. Please upload a completed version of your questionnaire as Supporting Information when you resubmit your manuscript.

2. Please amend your detailed Financial Disclosure statement. This is published with the article. It must therefore be completed in full sentences and contain the exact wording you wish to be published.

1. Please clarify all sources of funding (financial or material support) for your study. List the grants (with grant number) or organizations (with url) that supported your study, including funding received from your institution.

2. State the initials, alongside each funding source, of each author to receive each grant.

3. State what role the funders took in the study. If the funders had no role in your study, please state: “The funders had no role in study design, data collection and analysis, decision to publish, or preparation of the manuscript.”

4. If any authors received a salary from any of your funders, please state which authors and which funders.

3. We do not publish any copyright or trademark symbols that usually accompany proprietary names, eg (R), (C), or TM  (e.g. next to drug or reagent names). Please remove all instances of trademark/copyright symbols throughout the text, including ® on page 7.

If the reviewer comments include a recommendation to cite specific previously published works, please review and evaluate these publications to determine whether they are relevant and should be cited. There is no requirement to cite these works unless the editor has indicated otherwise.

Additional Editor Comments (if provided):

This is an important report on controlling viral hepatitis and, in particular, hepatitis C (HCV) in Africa. The report is well written and is also a good example of how to conduct feasibility studies. The authors managed to set a clear and moving background. However, there are a few issues that must be addressed.

Issues:

1. Please indicate how the 2000 sample size was determined.

2. Line 132 is a bit unclear. Can it be rewritten?

3. About the acceptability, it needs to be recognized that this instrument has never been tested in this population before. The EFA and CFA results are good, but it is not a guarantee that this will work forever.

The continuous acceptability score is used/analysed in two ways. One is the study of distribution using histograms and boxplots. The other one is through a dichotomization of the acceptability and use it as an outcome for the binary logistic regression.

• For the study of distribution, as in figure 3, I would suggest a cumulative counts plot. On such plot, you could put counts on the left y-axis, and percentages on the right y-axis. Heights are easier to read than histogram areas.

• About the “least 10% of acceptability” concept. Can we call this the lowest decile acceptability score?

• Figure 4 is successfully to show the distribution of the acceptability score for a few key variables. However, because it is based on quartiles, it fails to tell us the proportion of observations below the line of the overall lowest decile. I suggest adding a table with such proportions.

4. About the cascade. Table 2 is accurate and quite informative. Figure 2 is a bit deceptive, because it suggests that the denominators for each step are accurate [which is not true for the last step, for example]. So I suggest i) to keep table 2 and ii) compute accurate proportions (in percentages) and add 95% confidence intervals for the cascade plot.

[Note: HTML markup is below. Please do not edit.]

Reviewers' comments:

Reviewer's Responses to Questions

**Comments to the Author**

1. Does this manuscript meet PLOS Global Public Health’s publication criteria? Is the manuscript technically sound, and do the data support the conclusions? The manuscript must describe methodologically and ethically rigorous research with conclusions that are appropriately drawn based on the data presented.

Reviewer #1: Yes

**********

2. Has the statistical analysis been performed appropriately and rigorously?

Reviewer #1: No

**********

3. Have the authors made all data underlying the findings in their manuscript fully available (please refer to the Data Availability Statement at the start of the manuscript PDF file)?

The PLOS Data policy requires authors to make all data underlying the findings described in their manuscript fully available without restriction, with rare exception. The data should be provided as part of the manuscript or its supporting information, or deposited to a public repository. For example, in addition to summary statistics, the data points behind means, medians and variance measures should be available. If there are restrictions on publicly sharing data—e.g. participant privacy or use of data from a third party—those must be specified.

Reviewer #1: No

**********

4. Is the manuscript presented in an intelligible fashion and written in standard English?

PLOS Global Public Health does not copyedit accepted manuscripts, so the language in submitted articles must be clear, correct, and unambiguous. Any typographical or grammatical errors should be corrected at revision, so please note any specific errors here.

Reviewer #1: Yes

**********

5. Review Comments to the Author

Please use the space provided to explain your answers to the questions above. You may also include additional comments for the author, including concerns about dual publication, research ethics, or publication ethics. (Please upload your review as an attachment if it exceeds 20,000 characters)

Reviewer #1: Definitely a timely article - contributing to the evidence base around HCV self-testing - an important further step in HCV elimination methods.

Overall, I thought the paper to be well-written and close to publication. However, there were some areas that I thought needed either amendment or possible additional analysis. In particular, I have some real concerns about the description of acceptability analysis.

Abstract:

- Clarify that use of HCVST is referring only to antibody testing (this applies to the paper throughout)

- I would clarify that analysis is only based on 1,995 valid participants.

Introduction:

- Perhaps just a quick sentence describing the nature of Nigeria's epidemic - i.e. is it generalised of population specific?

Methods:

- IMPORTANT: Needs to be more information about participant recruitment. The paper at times implies that the large sample size equates to high acceptability - but there is no information about the refusal rate. This would actually be a critical aspect of determining acceptability of self-testing. If you recruited 2,000 participants - but an additional 2,000 refused to participate (for example) - this would suggest self-testing is acceptable to only 50% of clients.

- IMPORTANT: Very little in methods about your qualitative interviewing - this needs expansion.

- IMPORTANT: There is very little satisfactory information about the acceptability questionnaire - and it's subsequent transformation into an acceptability score using factor analysis. Currently, it reads as if the audience is simply supposed to trust the authors that all methods were satisfactory, and that - therefore - results are valid. You don't specify which questions were included in factor analysis, or if it was appropriate to score each item similarly or to aggregate scores. You also don't indicate if the questions were a previously developed tool or something developed specifically for the study - or that it's even available in the supplementary data. This almost feels like a separate paper, so that your method of transforming the acceptability survey can be more properly peer reviewed.

- IMPORTANT: For you acceptability analysis, how did you determine that 10% equals 'unacceptable'? Shouldn't people with only a 20% acceptability score be considered broadly non-accepting?? Also, considering all the noted limitations and collinearity issues with site - does it even make sense to compare by site?

- How did you determine the 2,000 sample size?

- The selection criteria is difficult to follow and should be clarified - perhaps with dot points?

- Specify that "blood-based" testing means finger prick?

- Again, need to clarify that testing is antibody, correct? Presuming also that RA information discussed testing procedures – i.e. what happened if the ST was positive and it’s interpretation?

- How many of each test did you have? Why did you not have enough tests so that any participant could choose whichever test they wanted for the entirety of the study period?

- Presuming drug abstention is not a requirement to treatment initiation?

- For the exposure variables - is 'education' referring to attendance or completion?

- You explain later in the paper that participants could be part of multiple KP categories - but probably good to explain in the "exposure variables" section.

Results:

- Non-considering possible refusal rates - your results suggest that of 2000 participants, essentially 100% completed the self-test? This is a remarkable outcome – particularly for the 849 participants who were unobserved!

- "Five participants 255 took HCVST kits home but did not return to complete the endline survey, therefore no demographic or clinical information was recorded for those participants, and they were excluded

from the analysis." Why wasn't demographic information collected at baseline, as would be usual?

- Suggest re-doing Figure 2 - removing the column for those 'tested' as this is inherently 100% and that every subsequent column is small by comparison. Add percentages to columns. Could consider comparing clinic types.

- Following on from methods comments regarding the acceptability data - you don't specify the number of participants falling within the purported "least 10%". The sentence describing qual data “Acceptability appeared to be more associated with clients characteristics than facility type…” - also suggests there are issues with your acceptability analysis methods.

- Did you consider differences across test type? In particular those who elected to conduct unobserved self-testing? What were the characteristics of these individuals?

- Sentence on page 18 “Observations about test type choice are thus descriptive only, not inferential” should probably be in the methods – following discussion of how tests ran out.

Discussion:

- How much do you think the demographic differences between facility-type clients impacted outcomes? For example – OSS clients are much younger and more males.

- OSS clients used more ‘blood’ tests. Did this have any impact on acceptability?

- The statement about PWID ‘surprise’ may be misplaced. How many PWID did you approach, who refused to participate? How does the number recruited compare against the OSS client list? Also, it’s 28% (PWID) compared to (31% MSM) – are you surprised about MSM?

- “Additionally, we did not assess whether the acceptability scale functioned equivalently across ART and OSS settings, so observed differences should be interpreted with that caveat.” - what does this statement mean? Is this another potential flaw of the acceptability scale?

- I'd include explanation of the SVR12 limitation somewhere higher in the paper.

**********

6. PLOS authors have the option to publish the peer review history of their article (what does this mean?). If published, this will include your full peer review and any attached files.

**Do you want your identity to be public for this peer review?**  If you choose “no”, your identity will remain anonymous but your review may still be made public.

For information about this choice, including consent withdrawal, please see our Privacy Policy.

Reviewer #1: **Yes:** Dr. Daniel O'Keefe

**********

[NOTE: If reviewer comments were submitted as an attachment file, they will be attached to this email and accessible via the submission site. Please log into your account, locate the manuscript record, and check for the action link "View Attachments". If this link does not appear, there are no attachment files.]

-->Figure Resubmissions:

While revising your submission, we strongly recommend that you use PLOS’s NAAS tool (https://ngplosjournals.pagemajik.ai/artanalysis) to test your figure files. NAAS can convert your figure files to the TIFF file type and meet basic requirements (such as print size, resolution), or provide you with a report on issues that do not meet our requirements and that NAAS cannot fix.

After uploading your figures to PLOS’s NAAS tool - https://ngplosjournals.pagemajik.ai/artanalysis, NAAS will process the files provided and display the results in the "Uploaded Files" section of the page as the processing is complete. If the uploaded figures meet our requirements (or NAAS is able to fix the files to meet our requirements), the figure will be marked as "fixed" above. If NAAS is unable to fix the files, a red "failed" label will appear above. When NAAS has confirmed that the figure files meet our requirements, please download the file via the download option, and include these NAAS processed figure files when submitting your revised manuscript.

# Author comment: Author response to Decision Letter 1

**DOI:** 10.1371/journal.pgph.0005567.r002

26 Jan 2026

Attachment Submitted filename: Cover_letter_reviewer_response_R1.doc

# Peer review: Decision Letter 1

**DOI:** 10.1371/journal.pgph.0005567.r003

**Authors:** Johanna Pruller

11 Mar 2026

PGPH-D-25-03540R1

Feasibility and acceptability of hepatitis C virus self-testing models among high-risk groups in Nasarawa, Nigeria; exploratory cross-sectional analysis of an implementation study.

PLOS Global Public Health

Dear Dr. Dunkley,

Thank you for submitting your manuscript to PLOS Global Public Health. After careful consideration, we feel that it has merit but does not fully meet PLOS Global Public Health’s publication criteria as it currently stands. Therefore, we invite you to submit a revised version of the manuscript that addresses the points raised during the review process.

==============================

The manuscript has been evaluated by one reviewer, and their comments are available below.

The reviewers have raised a number of concerns that need attention. Could you please revise the manuscript to carefully address the concerns raised? Specifically, the reviewer has raised concerns regarding the bespoke acceptability measure used in this study. Please carefully discuss why prior validity testing was not performed and how this may impact the results obtained. The lack of validity testing is concerning, and would need to be in depth explained and discussed in the limitations of this study.

==============================

Please submit your revised manuscript by Apr 25 2026 11:59PM. If you will need more time than this to complete your revisions, please reply to this message or contact the journal office at globalpubhealth@plos.org. When you're ready to submit your revision, log on to https://www.editorialmanager.com/pgph/ and select the 'Submissions Needing Revision' folder to locate your manuscript file.

Please include the following items when submitting your revised manuscript:

- A letter that responds to each point raised by the editor and reviewer(s). You should upload this letter as a separate file labeled 'Response to Reviewers'.
- A marked-up copy of your manuscript that highlights changes made to the original version. You should upload this as a separate file labeled 'Revised Manuscript with Track Changes'.
- An unmarked version of your revised paper without tracked changes. You should upload this as a separate file labeled 'Manuscript'.

Guidelines for resubmitting your figure files are available below the reviewer comments at the end of this letter.

We look forward to receiving your revised manuscript.

Kind regards,

Johanna Pruller, Ph.D.

PLOS Staff Editor

PLOS Global Public Health

**Journal Requirements:**

If the reviewer comments include a recommendation to cite specific previously published works, please review and evaluate these publications to determine whether they are relevant and should be cited. There is no requirement to cite these works unless the editor has indicated otherwise.

**Additional Editor Comments (if provided):**

[Note: HTML markup is below. Please do not edit.]

Reviewers' comments:

Reviewer's Responses to Questions

**Comments to the Author**

1. If the authors have adequately addressed your comments raised in a previous round of review and you feel that this manuscript is now acceptable for publication, you may indicate that here to bypass the “Comments to the Author” section, enter your conflict of interest statement in the “Confidential to Editor” section, and submit your "Accept" recommendation.

Reviewer #1: All comments have been addressed

**********

2. Does this manuscript meet PLOS Global Public Health’s publication criteria? Is the manuscript technically sound, and do the data support the conclusions? The manuscript must describe methodologically and ethically rigorous research with conclusions that are appropriately drawn based on the data presented.

Reviewer #1: Yes

**********

3. Has the statistical analysis been performed appropriately and rigorously?

Reviewer #1: I don't know

**********

4. Have the authors made all data underlying the findings in their manuscript fully available (please refer to the Data Availability Statement at the start of the manuscript PDF file)?

The PLOS Data policy requires authors to make all data underlying the findings described in their manuscript fully available without restriction, with rare exception. The data should be provided as part of the manuscript or its supporting information, or deposited to a public repository. For example, in addition to summary statistics, the data points behind means, medians and variance measures should be available. If there are restrictions on publicly sharing data—e.g. participant privacy or use of data from a third party—those must be specified.

Reviewer #1: Yes

**********

5. Is the manuscript presented in an intelligible fashion and written in standard English?

PLOS Global Public Health does not copyedit accepted manuscripts, so the language in submitted articles must be clear, correct, and unambiguous. Any typographical or grammatical errors should be corrected at revision, so please note any specific errors here.

Reviewer #1: Yes

**********

6. Review Comments to the Author

Please use the space provided to explain your answers to the questions above. You may also include additional comments for the author, including concerns about dual publication, research ethics, or publication ethics. (Please upload your review as an attachment if it exceeds 20,000 characters)

Reviewer #1: - Authors have responded to my comments

- Given the noted limitation of not being able to demonstrate comprehensive acceptability (without refusal rates) – it’s advised to further review the paper for instances of potential overstatement of findings. For example, on page 20. “…while both…facilities demonstrated high engagement…” – 1,000 clients is definitely a lot, but without the context of how many overall clinic clients there are, and how many may have refused, it would be prudent to temper the statements slightly.

- Importantly, the additional information provided by the authors about their bespoke acceptability measure gives me greater reservations – considering it has no prior validity testing. This is not addressed in the limitations and definitely should be.

**********

7. PLOS authors have the option to publish the peer review history of their article (what does this mean?). If published, this will include your full peer review and any attached files.

**Do you want your identity to be public for this peer review?**  If you choose “no”, your identity will remain anonymous but your review may still be made public.

For information about this choice, including consent withdrawal, please see our Privacy Policy.

Reviewer #1: **Yes:** Daniel O'Keefe

**********

[NOTE: If reviewer comments were submitted as an attachment file, they will be attached to this email and accessible via the submission site. Please log into your account, locate the manuscript record, and check for the action link "View Attachments". If this link does not appear, there are no attachment files.]

-->Figure Resubmissions:

While revising your submission, we strongly recommend that you use PLOS’s NAAS tool (https://ngplosjournals.pagemajik.ai/artanalysis) to test your figure files. NAAS can convert your figure files to the TIFF file type and meet basic requirements (such as print size, resolution), or provide you with a report on issues that do not meet our requirements and that NAAS cannot fix.

After uploading your figures to PLOS’s NAAS tool - https://ngplosjournals.pagemajik.ai/artanalysis, NAAS will process the files provided and display the results in the "Uploaded Files" section of the page as the processing is complete. If the uploaded figures meet our requirements (or NAAS is able to fix the files to meet our requirements), the figure will be marked as "fixed" above. If NAAS is unable to fix the files, a red "failed" label will appear above. When NAAS has confirmed that the figure files meet our requirements, please download the file via the download option, and include these NAAS processed figure files when submitting your revised manuscript.

# Author comment: Author response to Decision Letter 2

**DOI:** 10.1371/journal.pgph.0005567.r004

10 Apr 2026

Attachment Submitted filename: Cover_letter_reviewer_response_R2_090426.docx

# Peer review: Decision Letter 2

**DOI:** 10.1371/journal.pgph.0005567.r005

**Authors:** Julia Robinson

24 May 2026

Feasibility and acceptability of hepatitis C virus self-testing models among high-risk groups in Nasarawa, Nigeria; exploratory cross-sectional analysis of an implementation study.

PGPH-D-25-03540R2

Dear Ms Dunkley,

We are pleased to inform you that your manuscript 'Feasibility and acceptability of hepatitis C virus self-testing models among high-risk groups in Nasarawa, Nigeria; exploratory cross-sectional analysis of an implementation study.' has been provisionally accepted for publication in PLOS Global Public Health.

Before your manuscript can be formally accepted you will need to complete some formatting changes, which you will receive in a follow up email. A member of our team will be in touch with a set of requests.

Please note that your manuscript will not be scheduled for publication until you have made the required changes, so a swift response is appreciated.

IMPORTANT: The editorial review process is now complete. PLOS will only permit corrections to spelling, formatting or significant scientific errors from this point onwards. Requests for major changes, or any which affect the scientific understanding of your work, will cause delays to the publication date of your manuscript.

If your institution or institutions have a press office, please notify them about your upcoming paper to help maximize its impact. If they'll be preparing press materials, please inform our press team as soon as possible -- no later than 48 hours after receiving the formal acceptance. Your manuscript will remain under strict press embargo until 2 pm Eastern Time on the date of publication. For more information, please contact globalpubhealth@plos.org.

Thank you again for supporting Open Access publishing; we are looking forward to publishing your work in PLOS Global Public Health.

Best regards,

Julia Robinson

Executive Editor

PLOS Global Public Health

***********************************************************

Reviewer Comments (if any, and for reference):

Reviewer's Responses to Questions

**Comments to the Author**

1. If the authors have adequately addressed your comments raised in a previous round of review and you feel that this manuscript is now acceptable for publication, you may indicate that here to bypass the “Comments to the Author” section, enter your conflict of interest statement in the “Confidential to Editor” section, and submit your "Accept" recommendation.

Reviewer #1: All comments have been addressed

**********

2. Does this manuscript meet PLOS Global Public Health’s publication criteria? Is the manuscript technically sound, and do the data support the conclusions? The manuscript must describe methodologically and ethically rigorous research with conclusions that are appropriately drawn based on the data presented.

Reviewer #1: Yes

**********

3. Has the statistical analysis been performed appropriately and rigorously?

Reviewer #1: Yes

**********

4. Have the authors made all data underlying the findings in their manuscript fully available (please refer to the Data Availability Statement at the start of the manuscript PDF file)?

The PLOS Data policy requires authors to make all data underlying the findings described in their manuscript fully available without restriction, with rare exception. The data should be provided as part of the manuscript or its supporting information, or deposited to a public repository. For example, in addition to summary statistics, the data points behind means, medians and variance measures should be available. If there are restrictions on publicly sharing data—e.g. participant privacy or use of data from a third party—those must be specified.

Reviewer #1: No

**********

5. Is the manuscript presented in an intelligible fashion and written in standard English?

PLOS Global Public Health does not copyedit accepted manuscripts, so the language in submitted articles must be clear, correct, and unambiguous. Any typographical or grammatical errors should be corrected at revision, so please note any specific errors here.

Reviewer #1: Yes

**********

6. Review Comments to the Author

Please use the space provided to explain your answers to the questions above. You may also include additional comments for the author, including concerns about dual publication, research ethics, or publication ethics. (Please upload your review as an attachment if it exceeds 20,000 characters)

Reviewer #1: Thank you to the authors for addressing my comments.

**********

7. PLOS authors have the option to publish the peer review history of their article (what does this mean?). If published, this will include your full peer review and any attached files.

**Do you want your identity to be public for this peer review?**  If you choose “no”, your identity will remain anonymous but your review may still be made public.

For information about this choice, including consent withdrawal, please see our Privacy Policy.

Reviewer #1: **Yes:** Dr. Daniel O'Keefe

**********
